# Older patients are still under-represented in clinical trials of Alzheimer’s disease

**DOI:** 10.1186/s13195-016-0201-2

**Published:** 2016-08-12

**Authors:** Rita Banzi, Paolo Camaioni, Mauro Tettamanti, Vittorio Bertele’, Ugo Lucca

**Affiliations:** 1 Laboratorio Politiche Regolatorie del Farmaco, IRCCS Istituto di Ricerche Farmacologiche Mario Negri, Via La Masa 19, Milan, 20156 Italy; 2 Laboratorio di Neuropsichiatria Geriatrica, IRCCS Istituto di Ricerche Farmacologiche Mario Negri, Via La Masa 19, Milan, 20156 Italy

**Keywords:** Age, Alzheimer’s disease, Clinical trials, Disease-modifying drugs, External validity, Inclusion criteria, Trial participants

## Abstract

**Background:**

The age gap between participants in trials and patients who could benefit from the drugs studied has been widely documented across different clinical areas. Patients with dementia included in clinical research are systematically younger than those in the general population. We examined the age gap between participants in recent clinical trials testing interventions for Alzheimer’s disease and epidemiological data.

**Methods:**

We systematically searched literature databases (MedLine, EMBASE, the Cochrane Library) and ClinicalTrials.gov from 2000 to July 2015 to retrieve clinical trials testing pharmacologic treatments for Alzheimer’s disease, other than cholinesterase inhibitors and memantine. We included ongoing and completed phase II/III randomized clinical trials, irrespective of their publication status. From each study reporting the participants’ ages, we extracted size of sample, mean age, and standard deviation, and estimated the proportions of participants in different age classes. The number of patients with Alzheimer’s disease by age class in the USA population was used for comparison.

**Results:**

We included 165 clinical trials testing almost 100 different compounds, which enrolled or planned to enroll about 74,300 participants. Seventy-nine of these trials, accounting for about 26,800 participants, reported the age of the participants. The weighted mean age was 73.6 years (standard deviation, 8.2). People younger than 80 years were highly represented in clinical trials (78 %), despite the fact that those aged 80 and older form the majority (72 %) of patients with Alzheimer’s disease. Only 8 % of clinical trial participants were 85 years or older.

**Conclusions:**

Patients enrolled in clinical trials on Alzheimer’s disease are far from being representative of actual distribution of the patients in the general population. Clinical research should not be designed and conducted overlooking the fact that the majority of individuals with Alzheimer’s disease are likely to be 80 or older.

**Electronic supplementary material:**

The online version of this article (doi:10.1186/s13195-016-0201-2) contains supplementary material, which is available to authorized users.

## Background

Between 1993 and 2003, four acetylcholinesterase inhibitors and memantine were licensed in the USA and Europe for the symptomatic treatment of Alzheimer’s disease [1, 2]. In the last few decades, research has shifted mainly towards putative disease-modifying agents. Although several candidate drugs have been tested in phase II and III trials, no new treatments have been licensed since 2002 in Europe and 2003 in the USA [3, 4]. Two reviews identified about 200 drug development failures for Alzheimer’s disease [5, 6].

Irrespective of the fact that these drugs did not reach the market in the end, it is important to investigate whether the participants enrolled in this large number of trials were representative of the general population of patients who would have received a drug if it had been marketed. In the late 1990s, Schneider and co-authors estimated the percentage of patients with Alzheimer’s disease in a general clinical population who might have been eligible for inclusion in two typical, industry-sponsored trials testing the efficacy of symptomatic drugs for Alzheimer’s disease [7]: only a small proportion (4.4–7.9 %) of the overall clinical population of Alzheimer’s disease patients would have been eligible for each of the two trials. Women were under-represented and the very few eligible patients were younger, better educated, and wealthier than the ineligible patients. This was confirmed by a recent review on the representativeness of patients included in trials on acetylcholinesterase inhibitors, which showed that participants were younger than real-life patients with Alzheimer’s disease and women were under-represented [8]. Given the large numbers of trials on Alzheimer’s disease in the last few decades, it seemed worth assessing the recent Alzheimer’s disease agenda.

The age gap between participants in trials and patients who could benefit from the study drugs has been widely documented across different clinical areas [9, 10]. People with dementia who are included in clinical research are systematically younger than patients from the general population: a review found a gap of about 8 years in the mean age [11]. However, the prevalence of Alzheimer’s disease and dementia increases considerably with age, being fairly low among people aged under 75 and much higher in the oldest groups [12, 13]. Considering the census population projection, the total number of people 65 years and older with Alzheimer’s disease in the USA is currently estimated at 5.1 million, 2.0 million of them aged 85 and older (39 %) and 0.8 million aged 65–74 years (16 %). In 2050, the total number of persons 65 years and older with Alzheimer’s disease may reach 13.8 million, with 7.0 million 85 or older (51 %) and 1.3 million 65–74 years (9 %) [14].

Whether patients included in clinical trials are representative of those who would be exposed to new drug treatments in clinical practice is, therefore, a major question. Patients in the general population may respond differently to a drug than might those meeting the stringent selection criteria of clinical trials, or may be exposed to unexpected harm. Geriatric societies and regulatory agencies have clearly stated that age should not be a barrier to participation in trials and that participants should reflect the population that will receive the drug once marketed [15]. Concern about under-representation of older participants is particularly relevant to conditions like dementia and Alzheimer’s disease, which mainly affect the oldest members of society.

This review examined whether the age of the participants in randomized clinical trials on Alzheimer’s disease reflects that of the people with the disease in the general population. We focused on the recent Alzheimer’s disease research agenda, i.e. pharmacological treatments evaluated after the introduction of the symptomatic drugs licensed in Europe and USA in the early 2000s, to add to previous reviews [8, 11].

## Methods

### Criteria for considering studies for review

We followed a systematic approach to develop the sample for this study. We sought clinical trials that evaluated one or more pharmacological interventions proposed to improve cognitive or functional outcomes in participants with a diagnosis of Alzheimer’s disease (any stage and diagnostic criteria). We included ongoing and completed phase II and III randomized clinical trials, irrespective of their publication status. We excluded studies on acetylcholinesterase and memantine because they had already been analyzed and our review focused on the most recent interventions for Alzheimer’s disease, aimed at slowing the natural progression of the disease. We excluded studies testing non-pharmacological interventions (e.g. herbal and natural products, dietary intervention, rehabilitation, cognitive-behavioral approaches), diagnostic tools (biomarkers), or interventions not addressing patients (e.g. caregivers). We also excluded phase I and I/II studies assessing toxicity or optimal doses. Finally, we excluded studies testing drugs to prevent the onset of Alzheimer’s disease in subjects with no or mild cognitive impairment.

### Identification of studies

To identify eligible studies, we systematically searched literature databases (MedLine, EMBASE, the Cochrane Library) and the trial registry ClinicalTrials.gov from 2000 to July 2015. We also searched the proceedings of the main international conferences in the field of Alzheimer’s disease during the same period. Additional file 1 reports details of search strategies.

### Data collection and analysis

One review author screened the title and abstract to assess eligibility. We considered this acceptable as the inclusion criteria were simple, broad, and quite straightforward. Selection was inclusive at this stage and aimed to exclude all records clearly irrelevant for the purpose of this review. A random sample of the abstracts (15 %) was then screened by a second reviewer to check the accuracy of the process. Two authors independently confirmed the eligibility and extracted the data using an *ad-hoc* extraction form. For each study included, we extracted the year of publication or registration, trial name and registration number; publication status (published, terminated, ongoing, etc.); countries, sponsors and type of funding (public or private); study design (cross-over, parallel, blinded); number of patients (actually enrolled or planned to be enrolled), inclusion and exclusion criteria (type of diagnostic criteria, age and Mini-Mental State Examination range at inclusion, stage of the disease, main exclusion criteria, and prohibited concomitant medications); experimental and control interventions; main mechanism of action; length of treatment and follow-up, and the primary outcome measure. We also extracted demographic variables, including age, sex, and years of education. We did not assess possible biases affecting the internal validity of trials, as our aim was to evaluate the representativeness of the population included, which affects the external validity of the trial results.

From the subset of studies that reported the age of the population included, we extracted the size of the population, mean age, and standard deviation. When median and quartiles were reported instead of mean and standard deviation, we calculate the mean from the average of the 25, 50 and 75 percentiles (obtaining, as expected, numbers very close to the reported medians); we calculated the standard deviation by multiplying the mean of the differences between quartiles and estimated mean by 1.5. We calculated the mean of the standard deviations of the studies included and assumed that this variability also applied to the studies that did not give enough details to calculate the standard deviation, e.g. those reporting age as mean (or median) and range (min–max). We considered it unfeasible to contact the authors or principal investigators to collect missing information.

The proportion of subjects in the different age classes was calculated assuming the age distribution was normal. We assumed a singly or doubly truncated normal distribution for the studies that set a lower or upper age limit, or both, in their inclusion criteria (e.g., patients up to 85 years of age). For each study, we used the mean and standard deviation to calculate the percentiles corresponding to the specified age classes, then multiplied the difference between these consecutive percentiles by the size of the population to obtain an estimate of the number of patients in the specified age classes. Two studies reported the mean and standard deviation and the distribution in some age classes, which meant that we could check the accuracy of this estimation: the concordance was roughly 90 %, suggesting that estimations and actual values were similar at least in the small sample available. For each study, we then estimated the numbers of participants in the following age classes: less than 65, 65–74, 75–84, and 85 years and older. We chose these classes to permit direct comparison with the number of Alzheimer’s disease patients in the US population [16]. Since the 10 year age class 75–84 comprises a particularly heterogeneous population, we split it into two 5 year classes (75–79 and 80–84) to permit more detailed comparison of the proportion of Alzheimer’s disease patients enrolled in clinical trials and in the general population. We used data from the Aging, Demographics, and Memory study ([17] and personal communication) and the Framingham study [18] as sources of prevalence data in these 5 year age classes.

## Results

**Fig. 1:**
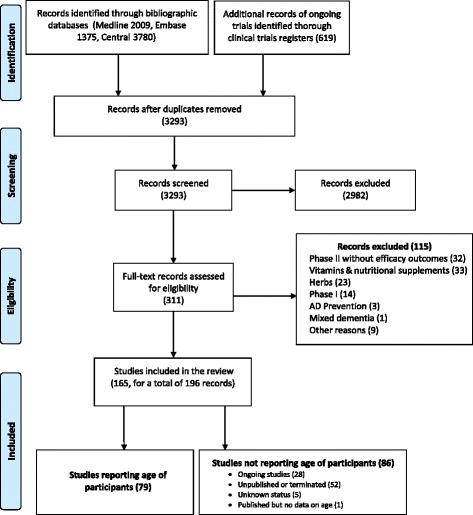
Flow chart of studies in this review. AD, Alzheimer’s disease

Database searches returned 3293 entries; 2982 were excluded by screening titles and abstracts and 311 were assessed for eligibility. Of these, 115 were excluded mainly because they were phase I or I/II trials, or phase II trials without efficacy endpoints as primary outcome, or were assessing vitamins, herbs or other natural products, or preventive strategies (see Fig. 1 and Additional file 2). We included the remaining 196 that reported information on 165 single clinical trials listed in Additional file 3. In all, these trials enrolled or planned to enroll about 74,300 participants.

At the time of data extraction, half the studies (80 out of 166) had published their results either in scientific papers or in trial registries, 28 were ongoing, 52 were terminated or ended but not published, and 4 were reported as ‘unknown’ in ClinicalTrials.gov. We could not retrieve further information on one additional study whose methods and analysis plan were published in 2002 [19]. Two published their trial protocols in medical journals [19, 20]. One-third were phase III trials.

We included trials on almost 100 different compounds, the majority proposed as anti-amyloid therapies, modulators of different neurotransmitter pathways (cholinergic, histaminergic, serotoninergic, etc.), or tau-protein modulators (Table 1). Several drugs were claimed to modify the natural progression of the disease by interfering with the pathogenic steps responsible for the clinical symptoms, including, for example, the deposition of extracellular amyloid beta plaques and intracellular neurofibrillary tangles, inflammation, oxidative damage, glucose and cholesterol metabolisms.

**Table 1:** Drugs tested in the clinical trials included in the review and mechanisms of actions

Class and proposed mechanism of action	Drug	Studies
Anti-amyloid		
Reduced amyloid beta aggregation or oligomerization	Tramiprosate (3APS)	3 phase III
Increased amyloid beta clearance, active immunotherapy	AN1792	1 phase II
	Sodium oligo-mannurarate	1 phase II, 1 phase III
Increased amyloid beta clearance, passive immunotherapy	Solanezumab	3 phase III
	Bapineuzumab	1 phase II, 4 phase III
	Gantenerumab	2 phase III
	BAN2401	1 phase II
	Crenezumab	1 phase II
	Aducanumab	2 phase III
	Immunoglobulin	1 phase II, 3 phase III
Increased amyloid beta clearance, other	Clioquinol (PBT-1)	1 phase II
Reduced amyloid beta production, gamma secretase inhibitor	Semagacestat	2 phase II, 2 phase III
Reduced amyloid beta production, gamma secretase modulator	Tarenflurbil	1 phase II, 2 phase III
Reduced amyloid beta production, beta secretase inhibitor	MK-8931	1 phase II/III, 1 phase III
	AZD3293 (LY3314814)	1 phase II/III
	E2609	1 phase II
Glycation end products receptor (RAGE) inhibitors	Azeliragon (TTP 488)	1 phase II, 1 phase III
Reduced amyloid beta	Nilvadipine	1 phase III
	Statins	1 phase II, 2 phase III
Anti-tau		
Aggregation inhibitor	TRx0237 (formerly TRx0014)	1 phase II, 2 phase III
	Tideglusib	1 phase II
Modulators of blood glucose		
	Insulins	4 phase II, 1 phase II/III
	Rosiglitazone	1 phase II, 3 phase III
	Pioglitazone	2 phase II
	AC 1204	2 phase II/III
Phosphodiesterase inhibitors		
Phosphodiesterase 9A	PF-04447943	1 phase II
	BI 409306	2 phase II
Phosphodiesterase 5	Udenafil	1 phase III
Phosphodiesterase 4	MK 0952	1 phase II
Histamine modulators		
	GSK239512	1 phase II
	MK0249	1 phase II
	ABT 288	1 phase II
	Nizatidine	1 phase ?
	SAR110894D	1 phase II
	S38093	2 phase II
Nicotine modulators		
Nicotinic acetylcholine receptor partial agonist	ABT-089	2 phase II
	RO5313534	1 phase II
	EVP-6124	1 phase II, 2 phase III
	Varenicline	1 phase II
	AZD3480	2 phase II
	AQW051	1 phase II
α-7 nicotinic acetylcholine receptor allosteric modulator	ABT-126	3 phase II
Muscarine modulators		
Allosteric modulator	MK 7622	1 phase II
Allosteric modulator	Lu25-109	1 study ?
Non-selective muscarinic acetylcholine receptor agonist	Talsaclidine	2 phase II
Serotonin modulators		
5-hydroxytryptamine 6 receptor antagonist	SB 742457	4 phase II
	Lu AE58054	1 phase II, 2 phase III
	SAM-531	1 phase II
5-hydroxytryptamine 1A receptor agonist	Xaliproden	2 phase III
	Paliroden^a^	1 phase II
5-hydroxytryptamine 1 receptor antagonist	Lecozotan	1 phase II, 1 phase II/III
5-hydroxytryptamine 4 receptor agonist	PRX-03140	2 phase II
Other central nervous system transmitter modulators		
Benzodiazepine receptor partial inverse agonist	AC-3933	1 phase II
Norepinephrine uptake inhibitor	Atomoxetine	1 phase II/III
NMDA receptor channel blocker	Neramexane	1 phase III
Monoamine uptake inhibitor	NS 2330	1 phase II
Adrenergic receptor antagonist	ORM-12741	1 phase II
Irreversible monoamine oxidase inhibitor	Rasagiline	1 phase II
GABA(B) receptor antagonist	SGS742	1 phase II
Glutamate receptor modulator	LY451395	1 phase II
Anti-inflammatory		
Non-steroidal anti-inflammatory drugs	Aspirin	1 phase III
	Indomethacin	1 phase III
	Ibuprofen	1 phase ?
	Nimesulide	1 phase II
	Rofecoxib	1 phase II/III, 1 phase ?
	Celecoxib	1 phase III
	Lornoxicam	1 phase II
Unknown mechanism	Hydroxychloroquine	1 phase III
Astrocyte activator	ONO-2506PO	1 phase II
	PYM50028	1 phase II
Hormones and analogs		
Antiprogestinic	Mifepristone	3 phase II
Corticosteroid	Prednisone	1 phase III
11-β-hydroxysteroid dehydrogenase type 1 inhibitor	ABT-384	1 phase II
Gonadotropin-releasing hormone analog	Leuprolide acetate	1 phase II
Estrogen receptor modulator	Raloxifene	1 phase II
	HRT	5 phase ?
Androgen receptor modulator	Dehydroepiandrosterone/testosterone	2 phase ?
Growth hormone secretagogue	MK0677	1 phase II
Other or several mechanisms		
c-kit (tyrosine kinase) inhibitor	Masitinib	1 phase II, 1 phase III
	Dimebon	2 phase II, 5 phase III
	IFNβ-alpha 2a	1 phase II
	IFNβ1a	1 phase II
	N-acetylcysteine	1 phase II
	Acetyl-L-carnitine	1 phase ?
Calcium antagonist	MEM 1003	1 phase II
Pleiotropic	ST101	2 phase II
	Doxycycline and rifampin	1 phase II, 1 phase III
	Cerebrolysin	4 phase II, 1 phase III
Somatostatin production enhancer	FK962	1 phase II
Unknown	VI-1121	1 phase II
	Idebenone	1 phase ?, 1 phase III
	Sodium oligo-mannurate	1 phase II
	RO4601522	1 phase II
	T-817MA	1 phase II

^a^mechanism not fully elucidated

The majority of trials evaluated a population with mild to moderate Alzheimer’s disease (132 of 165, 80 %), defined as probable (127 of 165, 77 %) according to the diagnostic criteria of the National Institute of Neurological and Communicative Disorders and Stroke (NINCDS) and the Alzheimer’s Disease and Related Disorders Association (ADRDA) [21] (73 of 165 studies, 44 %) or to a combination of NINCDS-ADRDA and Diagnostic and Statistical Manual of Mental Disorders criteria (28 of 165 studies, 17 %). Two-thirds were conducted in the USA, Canada, and Europe and 23 % were global trials involving clinical centers on at least three continents.

The eligibility criteria in terms of age varied. The lower age limit was usually 50 years, though seven set it at 40 [22–25] or 45 years [26–28]. The upper limit was between 85 and 90 years in 42 % of studies. No upper age limit was set in 44 % of the studies, meaning that, for example, any participant older than 50 years could be enrolled.

**Fig. 2:**
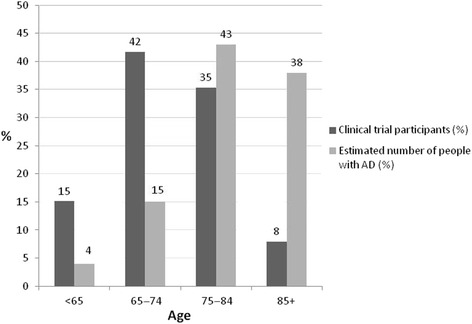
Distribution by age. Data from clinical trials and the estimated number of patients with Alzheimer’s disease in the USA in 2015 (source of prevalence data: [16]). AD, Alzheimer’s disease

Of the 165 studies, 79 provided data on the age of the population enrolled, for a total of 26,845 participants (Additional file 4). This subset of trials was used to calculate the proportion of subjects in the different age classes. The weighted mean age was 73.6 years (standard deviation 8.2) while the estimated mean age of the comparator population was 82 years [14]. Only 8 % of participants in clinical trials were 85 years or older, while most were in the age classes 75–84 (35 %) and 65–74 (42 %). Excluding phase II trials did not substantially change the proportion of patients in each class (younger than 65, 15 %; 65–74, 41 %; 75–84, 36 %; 85 years or older, 9 %). These figures do not correspond to the actual distribution of patients with Alzheimer’s disease in the general population. In 2015, 81 % of people with Alzheimer’s disease were 75 years or older and 38 % 85 years and older (Fig. 2) [16]. These proportions may well reach 90 % and 51 % by 2050 [14].

**Fig. 3:**
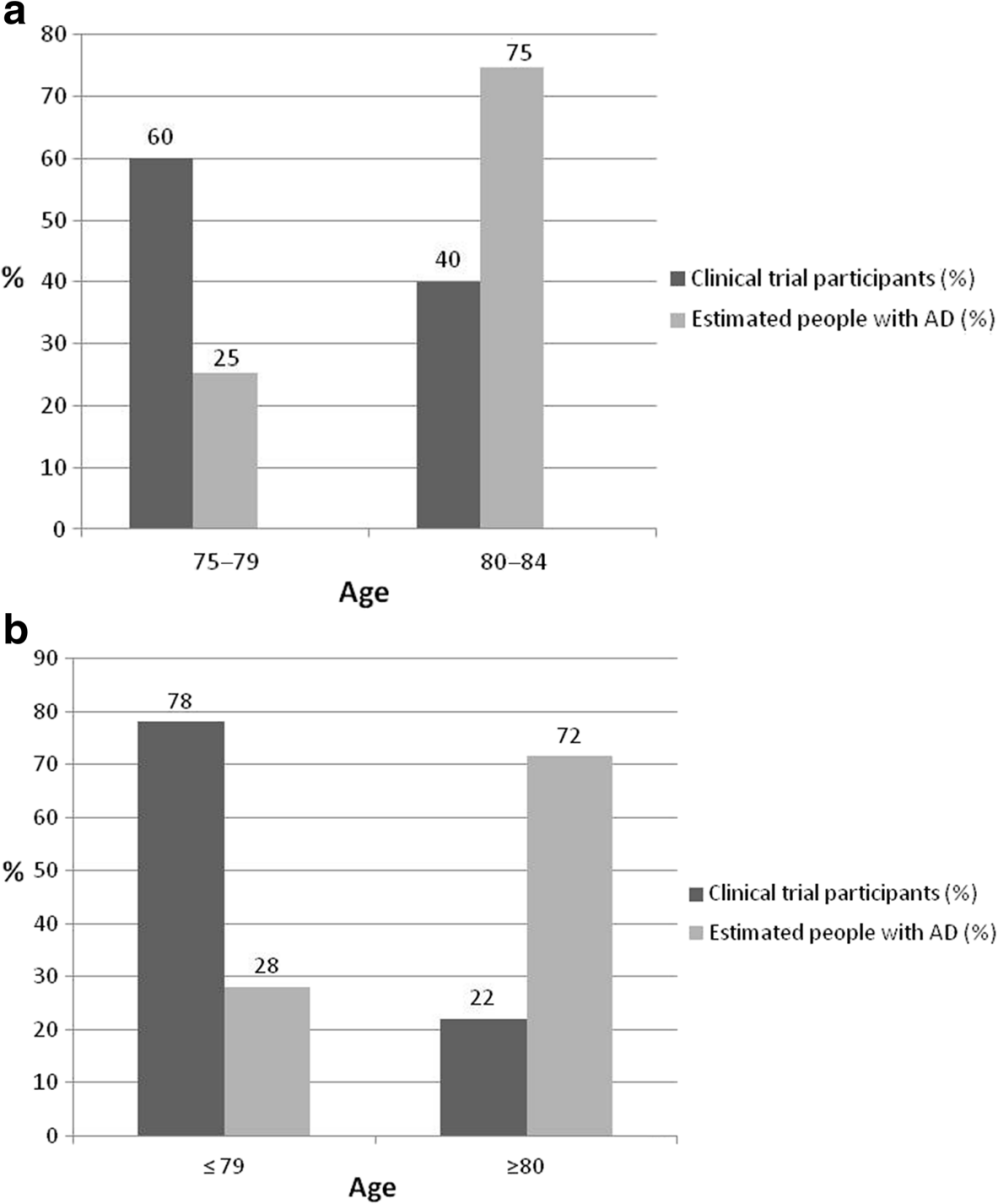
**a** Distribution in the 75–79 and 80–84 age classes. Data from clinical trials and the estimated number of patients with Alzheimer’s disease in the USA (source of prevalence data: [17, 18]). **b** Age distribution below and above age 80 years. Comparison of data from clinical trials and the estimated number of patients with Alzheimer’s disease in the USA (source of prevalence data: [17, 18]). AD, Alzheimer’s disease

The breakdown of the 75–84 age class showed that the proportion of clinical trial participants 80–84 years old accounted for more than one-third of the total (40 % 80–84 vs. 60 % 75–79, Fig. 3a). Alzheimer’s disease patients aged 80–84 years amount to three-quarters of those in the general population (75 % 80–84 versus 25 % 75–79, Fig. 3a) [17, 18]. More generally, the proportion of people 80 years and older reaches only about one-third of participants in clinical trials, far fewer than the numbers with Alzheimer’s disease of this age in the general population (Fig. 3b).

## Discussion

We analyzed recent trials of pharmacological treatments for Alzheimer’s disease and found that the age of the participants does not reflect the actual distribution of patients in the general population. Individuals older than 84 years contribute greatly to the number of people with Alzheimer’s disease (currently 38 %), a proportion that is expected to grow further in the coming decades (to about 51 % in 2050) [14]. Only 8 out of 100 trial participants in the sample fall into this class. In contrast, 15 % of patients enrolled were younger than 65 years, so they cannot even be defined as old. People younger than 80 are too widely represented in clinical trials, therefore, despite the fact that those aged 80 and more are the large majority with Alzheimer’s disease (72 %).

Involving old persons in clinical research is often considered too challenging because coexisting diseases, functional disability, and multi-drug treatments are common and may confound trial results. Some authors suggest that the differences between participants in observational and clinical studies and target population are magnified when research involves older adults and that this may reduce the generalizability [29]. Moreover, ethical issues linked to the inclusion of a vulnerable population have to be considered. However, any real or alleged obstacle to research participation should be weighed against the fact that excluding patients who represent the largest part of the population with the disease may lead to misinterpretation in the conclusions about the underlying neuropathology, diagnostic methods, or therapy [11].

In the case of Alzheimer’s disease and other conditions that overwhelmingly affect old people, the inclusion of representative samples of patients in trials testing innovative pharmacological approaches and new drugs should be the rule. Older people have different pharmacokinetics and pharmacodynamics from people in their 60s or 70s [30]. These diversities may translate into different efficacy and safety profiles; benefits as well as harms may be under- or overestimated, depending on the disease expression, and must be assessed before new drugs are used. The results of robust and valid trials (internal validity) are also only clinically useful if they are relevant to a definable patient population that should represent the target group of patients for the intervention under study. This concept, known as external validity, applicability, or generalizability, is often neglected, especially in industry-sponsored research [10], which selects the best possible experimental conditions to highlight the efficacy of new treatments, often disregarding their effectiveness in the real world.

Trial participants should reflect the actual distribution of individuals with Alzheimer’s disease in terms of other important aspects too, such as co-morbidities, cultural and educational background, and frailty. These variables may threaten the external validity of clinical trials in Alzheimer’s disease as well as age.

In clinical practice too, subjects treated with the marketed drugs are a small proportion of those with Alzheimer’s disease. For instance, the largest proportions of patients treated with acetylcholinesterase inhibitors were in the younger age groups, with a steep drop with age: from 55 % at 60–69 years to 19 % at 80–84 years [31]. This may be because of perceived low effectiveness of these drugs by both the caregiver and the physician, difficulties with diagnoses of dementia, significant concurrent pathologic abnormalities, adverse drug reactions, or even a fatalistic acceptance of the condition. It is unlikely, however, that the reasons behind these therapeutic decisions are linked to strict application of evidence generated in a younger population.

Our analysis has several advantages. It focuses on the most recent research on Alzheimer’s disease and adds to previous reviews of trials of older interventions [8, 11]. We retrieved published and unpublished trials on putative disease-modifying agents for Alzheimer’s disease using a systematic approach and based our analysis on a large sample of studies, covering different pharmacological treatments, mechanisms of action, trial sponsors, and countries. The sample can be considered highly representative of current research in the field of Alzheimer’s disease, even if we could not retrieve reports of about one-third of the studies included. While it is uncertain whether the inclusion of these trials would have changed our findings, it is worrying that so many trial reports are still inaccessible.

One further limitation is the lack of reporting on the proportions of patients per age class in clinical trials. The age of the study population is generally described using mean and standard deviation or median and range. Thus, we could only estimate the proportion of patients in each age class assuming a specific underlying distribution, i.e., taking a normal distribution for reference. This approximation, however, is likely to have had only a limited impact on the overall conclusion of the review, given the very large age differences between trial participants and patients in the general population. To understand the applicability of the evidence from clinical trials to oldest people better, complete reporting of the age distribution of the patients is a major, necessary step forward.

Several studies reported prevalence data on dementia [32], while a few focused specifically on Alzheimer’s disease. To estimate the number of people with Alzheimer’s disease by age class in the general population, we used prevalence data from different sources. This is because the most recent figures we found indicated only the number of people with Alzheimer’s disease by 10 year classes of age [14]. Thus, to retrieve Alzheimer’s disease prevalence estimates by 5 year classes, we referred to published and unpublished data from previous cohort studies [17, 18].

Finally, we compared the ages of participants in clinical trials only with the number of people with Alzheimer’s disease in the USA because more than half the trials involved clinical centers in North America. Comparison with data from other population studies could be of interest. However, our estimates are likely to be conservative as taking as reference the populations of European countries, Australia, Canada, and Japan, where the proportions of older subjects (older than 65) and oldest subjects (older than 80 or 85) are even larger than in the USA, would have led to a more dramatic difference.

## Conclusions

Lack of generalizability is an important obstacle to correct evidence-based practice. Taking USA data for comparison, the over-representation of younger old people (below 80 years) in clinical trials is clear. Clinical trials are the most reliable method of determining the effects of treatments, and meeting the diverse needs of patients, prescribers, regulators, and payers. They should collect data on clinically meaningful outcomes measured in populations representing the patients for whom the drug will eventually be licensed. It is to be hoped that new effective drugs will be available in the near future to treat Alzheimer’s disease, which is not only a devastating disorder, but also has a major social and economic impact [33]. Clinical research should not be designed and conducted so that it ignores the vast majority of Alzheimer’s disease patients aged 80 or 85 years and older [13, 14].

## Additional files

Additional file 1: Details of the search strategies. (DOCX 15 kb) Additional file 2: list of excluded studies. (DOCX 129 kb) Additional file 3: List of studies included. (DOCX 34 kb) Additional file 4: Main characteristics of trials included in the age analyses (79). (DOCX 30 kb)
