# Supplementary material for: Older patients are still under-represented in clinical trials of Alzheimer’s disease
Source: Alzheimers Res Ther. 2016 Aug 12;8:32. doi: 10.1186/s13195-016-0201-2 (PMC4982205; doi:10.1186/s13195-016-0201-2)
Supplement: Additional file 1: — Details of the search strategies. (DOCX 15 kb) [file 13195_2016_201_MOESM1_ESM.docx]

**Additional file 1: search strategies**

MedLine

((Alzheimer AND trial* OR ((( "Alzheimer Disease/drug therapy"[Mesh] OR "Alzheimer Disease/therapy"[Mesh] )) AND ((Clinical Trial, Phase II[ptyp] OR Clinical Trial, Phase III[ptyp] OR Randomized Controlled Trial[ptyp] OR Controlled Clinical Trial[ptyp])))

Embase

#8 #6 OR #7

#7 'alzheimer disease'/exp/mj/dm_dt,dm_th AND ([controlled clinical trial]/lim OR [randomized controlled trial]/lim) AND [embase]/lim AND [1995-2015]/py

#6 #4 AND #5

#5 #2 OR #3

#4 'alzheimer disease'/exp/mj/dm_dt,dm_th AND [embase]/lim AND [1995-2015]/py

#3 'phase 3 clinical trial'/exp AND [embase]/lim AND [1995-2015]/py

#2 'phase 2 clinical trial'/exp AND [embase]/lim AND [1995-2015]/py

The Cochrane Library

#1 Alzheimer:ti (Word variations have been searched)

#2 Alzheimer:ab

#3 #1 or #2

#4 #3 not PUBMED

#5 #4 not embase

#6 #5 Publication Year from 1995 to 2015

ClinicalTrials.gov

Interventional Studies | alzheimer | Phase 2, 3, 4 | received from 01/01/2000 to 10/07/2015

International conferences

Alzheimer's Association International Conference,

Clinical Trials on Alzheimer's Disease,

Springfield Symposium
