# Supplementary material for: Older patients are still under-represented in clinical trials of Alzheimer’s disease
Source: Alzheimers Res Ther. 2016 Aug 12;8:32. doi: 10.1186/s13195-016-0201-2 (PMC4982205; doi:10.1186/s13195-016-0201-2)
Supplement: Additional file 2: — list of excluded studies. (DOCX 129 kb) [file 13195_2016_201_MOESM2_ESM.docx]

**Additional file 2: list of excluded studies**

| **Main Reason for exclusion** | **Number of record (References)** |
| --- | --- |
| Phase II trials without primary efficacy outcomes | 32 ^1-32^ |
| Trials testing vitamins/nutritional supplements | 33 ^33-65^ |
| Trials testing herbal interventions | 23 ^66-88^ |
| Phase I trials | 4 ^89-102^ |
| Alzheimer's disease Prevention | 3 ^103-105^ |
| Mixed dementia | 1 ^106^ |
| Other reasons | 9 ^107-115^ |
| **Total** | **115** |

**References**

1. Endres K, Fahrenholz F, Lotz J, et al. Increased CSF APPs-alpha levels in patients with Alzheimer disease treated with acitretin. *Neurology*. 2014;**83**:1930-5.

2. Dodel R, Rominger A, Bartenstein P, et al. Intravenous immunoglobulin for treatment of mild-to-moderate Alzheimer's disease: a phase 2, randomised, double-blind, placebo-controlled, dose-finding trial. *Lancet Neurol*. 2013;**12**:233-43.

3. Rinne JO, Brooks DJ, Rossor MN, et al. 11C-PiB PET assessment of change in fibrillar amyloid-beta load in patients with Alzheimer's disease treated with bapineuzumab: a phase 2, double-blind, placebo-controlled, ascending-dose study. *Lancet Neurol*. 2010;**9**:363-72.

4. Lannfelt L, Blennow K, Zetterberg H, et al. Safety, efficacy, and biomarker findings of PBT2 in targeting Abeta as a modifying therapy for Alzheimer's disease: a phase IIa, double-blind, randomised, placebo-controlled trial. *Lancet Neurol*. 2008;**7**:779-86.

5. Aisen PS, Saumier D, Briand R, et al. A Phase II study targeting amyloid-beta with 3APS in mild-to-moderate Alzheimer disease. *Neurology*. 2006;**67**:1757-63.

6. Bayer AJ, Bullock R, Jones RW, et al. Evaluation of the safety and immunogenicity of synthetic Abeta42 (AN1792) in patients with AD. *Neurology*. 2005;**64**:94-101.

7. Simons M, Schwarzler F, Lutjohann D, et al. Treatment with simvastatin in normocholesterolemic patients with Alzheimer's disease: A 26-week randomized, placebo-controlled, double-blind trial. *Ann Neurol*. 2002;**52**:346-50.

8. Koch HJ, Szecsey A. A randomized controlled trial of prednisone in Alzheimer's disease. *Neurology*. 2000;**55**:1067.

9. Kennelly SP, Abdullah L, Paris D, et al. Demonstration of safety in Alzheimer's patients for intervention with an anti-hypertensive drug Nilvadipine: Results from a 6-week open label study. *International Journal of Geriatric Psychiatry*. 2011;**26**:1038-45.

10. Aisen PS, Briand R, Saumier D, Laurin J, Duong A, Garceau D. Targeting amyloid with tramiprosate in patients with mild-to-moderate Alzheimer disease. *Progress in Neurotherapeutics and Neuropsychopharmacology*. 2008;**3**:111-25.

11. Anon. [Public title] Study evaluating ACC-001 in Japanese patients with mild to moderate Alzheimer's disease; [Official title] A phase IIa, multicenter, randomized, third-party nnblinded, adjuvant and placebo controlled, safety, tolerability, and immunogenicity trial of ACC-001 and QS-21 adjuvant in Japanese subjects with mild to moderate Alzheimer's disease. Wyeth Clinical Trial Listings [wwwwyethcom/ClinicalTrialListings]; 2008.

12. Anon. [Public title] Long term extension study evaluating ACC-001 in subjects with mild to moderate Alzheimer's disease; [Scientific title] A phase IIa, multicenter, randomized, third-party unblinded, long-term extension study to determine safety, tolerability, and immunogenicity of ACC-001 with and without QS-21 adjuvant in subjects with mild to moderate Alzheimer's disease. ClinicalTrialsgov [<http://clinicaltrialsgov];> 2009.

13. Anon. [Public title] Safety, tolerability, and immunogenicity study of ACC-001 in subjects with mild to moderate Alzheimer's disease in Japan; [Scientific title] A phase IIA, multicenter, randomized, third-party unblinded, adjuvant and placebo controlled, safety, tolerability, and immunogenicity trial of ACC-001 and QS-21 adjuvant in Japanese patients with mild to moderate Alzheimer's disease. ClinicalTrialsgov [<http://clinicaltrialsgov];> 2009.

14. Anon. [Public title] Study evaluating safety, tolerability, and immunogenicity of ACC-001 in subjects with Alzheimer's disease; [Scientific title] Multicenter, randomized, multiple dose, safety, tolerability, and immunogenicity trial of ACC-001 in subjects with mild to moderate Alzheimer's disease. ClinicalTrialsgov [<http://clinicaltrialsgov];> 2009.

15. Nct. A trial of sk-pc-b70m in mild to moderate Alzheimer's disease or dose finding study to assess the efficacy and safety of sk-pc-b70m in patients with mild to moderate Alzheimer's disease. ClinicalTrialsgov [<http://clinicaltrialsgov];> 2007.

16. Anon. [Public title] 4 week, safety and tolerability study in patients with mild to moderate Alzheimer's disease (ROBIN); [Scientific title] Safety, tolerability and pharmacokinetics of 3 dose regimens of AZD1446 vs. placebo as an add-on treatment to donepezil: a multi-centre, double-blind, randomised, placebo controlled, parallel group phase IIa study in patients with mild to moderate Alzheimer's disease during 4 weeks of treatment. ClinicalTrialsgov [<http://clinicaltrialsgov];> 2009.

17. Anon. [Public title] A study to determine the clinical safety/tolerability and exploratory efficacy of EHT 0202 as adjunctive therapy to acetylcholinesterase inhibitor in mild to moderate Alzheimer's disease EHT0202/002; [Scientific title] A pilot, randomized, double-blind, placebo-controlled, parallel group, multicentre, phase IIA study to determine the clinical safety/tolerability and exploratory efficacy of EHT 0202 (40 and 80 mg Bid) as adjunctive therapy to acetylcholinesterase inhibitor over a 3-month period in ambulatory patients suffering from mild to moderate Alzheimer's disease (EHT 0202/002 Protocol). ClinicalTrialsgov [<http://clinicaltrialsgov];> 2008.

18. Anon. [Public title] Study of octagam 10% on the treatment of mild to moderate Alzheimer's patients; [Official title] Prospective 24-week, double-blind, randomized, multicenter, placebo-controlled study evaluating safety and change in surrogate parameters after treatment with increasing dosages of intravenous immunoglobulin (IGIV) in mild to moderate Alzheimer's disease. ClinicalTrialsgov [<http://clinicaltrialsgov];> 2008.

19. Anon. Safety and efficacy study of abt-089 in adults with mild to moderate Alzheimer's disease or a randomized, double-blind, placebo-controlled study using a bayesian adaptive design to evaluate the efficacy and safety of abt-089 in subjects with mild-to-moderate Alzheimer's disease on stable doses of acetylcholinesterase inhibitors. ClinicalTrialsgov [<http://clinicaltrialsgov];> 2007.

20. Anon. [Public title] A long term extension study evaluating ACC-001 in subjects with mild to moderate Alzheimer's disease; [Scientific title] A phase IIa, multicenter, randomized, third-party unblinded, long-term extension study to determine safety, tolerability, and immunogenicity of ACC-001 with and without QS-21 adjuvant in subjects with mild to moderate Alzheimer's disease. ClinicalTrialsgov [<http://clinicaltrialsgov];> 2009.

21. Anon. Tolerability and Primary Efficacy of CX516 in Alzheimer's Disease. NIH Clinical Research Studies; 2005.

22. Anon. Multicenter, randomized, third-party unblinded, multiple ascending dose, safety, tolerability, and immunogenicity trial of acc-001 in subjects with mild to moderate Alzheimer's disease. ClinicalTrialsgov [<http://clinicaltrialsgov];> 2008.

23. GlaxoSmithKline. A Double-Blind, Randomised, Placebo-Controlled, Parallel-Group Study to Investigate the Effects of Rosiglitazone (Extended Release Tablets) on Cerebral Glucose Utilisation and Cognition in Subjects With Mild to Moderate Alzheimer's Disease (AD). ClinicalTrialsgov [<http://clinicaltrialsgov];> 2006.

24. Anon. Pilot Study of Immunomodulatory versus Antiinflammatory Therapy in Alzheimer's Disease. ClinicalTrialsgov [<http://clinicaltrialsgov];> 2004.

25. Rossor MN. A randomised double blind safety tolerability and pilot efficacy study of AN1792 (QS-21) in patients with mild to moderate Alzheimer's disease. National Research Register; 2003.

26. A Randomized, Double-Blind, Placebo-Controlled, Safety, Tolerability, Pharmacokinetics, Pharmacodynamics Trial of Multiple Ascending Fixed Doses of SRA-333 in Subjects With Mild to Moderate Alzheimer’s Disease. ClinicalTrialsgov [<http://clinicaltrialsgov];> 2005.

27. Anon. Multicenter, randomized, multiple dose, safety, tolerability, and immunogenicity trial of acc-001 in subjects with mild to moderate Alzheimer's disease. ClinicalTrialsgov [<http://clinicaltrialsgov];> 2007.

28. GlaxoSmithKline. A double-blind, randomized, placebo-controlled, parallel-group study to investigate the effects of rosiglitazone (extended release tablets) on cerebral glucose utilization and cognition in subjects with mild to moderate Alzheimer's disease (AD). ClinicalTrialsgov [<http://clinicaltrialsgov];> 2005.

29. SK Chemicals Co. L. A Confirmatory Trial of SK-PC-B70M in Mild to Moderate Alzheimer's Disease, https://clinicaltrials.gov/ct2/show/NCT01249196. ClinicalTrialsgov [<http://clinicaltrialsgov];> 2010.

30. ForuM Pharmaceuticals Inc. Study of the Safety of Two Doses of Investigational Study Drug EVP-6124 in Subjects With Alzheimer's Disease Currently Receiving Memantine, https://clinicaltrials.gov/ct2/show/NCT01969123. ClinicalTrialsgov [<http://clinicaltrialsgov];> 2013.

31. National Institute of Aging. Pilot Clinical Trial of Exendin-4 in Alzheimer's Disease, https://clinicaltrials.gov/ct2/show/NCT01255163. ClinicalTrialsgov [<http://clinicaltrialsgov];> 2010.

32. CoMentis. GTS21-201 for Alzheimer Disease:GTS-21 Administered Daily for 28 Days to Participants With Probable Alzheimer’s Disease, https://clinicaltrials.gov/ct2/show/NCT00414622. 2006.

33. van der Zwaluw NL, Dhonukshe-Rutten RA, van Wijngaarden JP, et al. Results of 2-year vitamin B treatment on cognitive performance: Secondary data from an RCT. *Neurology*. 2014;**83**:2158-66.

34. Olde Rikkert MG, Verhey FR, Blesa R, et al. Tolerability and Safety of Souvenaid in Patients with Mild Alzheimer's Disease: Results of Multi-Center, 24-Week, Open-Label Extension Study. *J Alzheimers Dis*. 2014.

35. de Waal H, Stam CJ, Lansbergen MM, et al. The effect of souvenaid on functional brain network organisation in patients with mild Alzheimer's disease: a randomised controlled study. *PLoS One*. 2014;**9**:e86558.

36. Dysken MW, Sano M, Asthana S, et al. Effect of vitamin E and memantine on functional decline in Alzheimer disease: the TEAM-AD VA cooperative randomized trial. *JAMA*. 2014;**311**:33-44.

37. Shinto L, Quinn J, Montine T, et al. A randomized placebo-controlled pilot trial of omega-3 fatty acids and alpha lipoic acid in Alzheimer's disease. *J Alzheimers Dis*. 2014;**38**:111-20.

38. Dysken MW, Guarino PD, Vertrees JE, et al. Vitamin E and memantine in Alzheimer's disease: clinical trial methods and baseline data. *Alzheimers Dement*. 2014;**10**:36-44.

39. Scheltens P, Twisk JW, Blesa R, et al. Efficacy of Souvenaid in mild Alzheimer's disease: results from a randomized, controlled trial. *J Alzheimers Dis*. 2012;**31**:225-36.

40. Annweiler C, Fantino B, Parot-Schinkel E, Thiery S, Gautier J, Beauchet O. Alzheimer's disease--input of vitamin D with mEmantine assay (AD-IDEA trial): study protocol for a randomized controlled trial. *Trials*. 2011;**12**:230.

41. Stein MS, Scherer SC, Ladd KS, Harrison LC. A randomized controlled trial of high-dose vitamin D2 followed by intranasal insulin in Alzheimer's disease. *J Alzheimers Dis*. 2011;**26**:477-84.

42. Kwok T, Lee J, Law CB, et al. A randomized placebo controlled trial of homocysteine lowering to reduce cognitive decline in older demented people. *Clin Nutr*. 2011;**30**:297-302.

43. Cornelli U. Treatment of Alzheimer's disease with a cholinesterase inhibitor combined with antioxidants. *Neurodegener Dis*. 2010;**7**:193-202.

44. Remington R, Chan A, Paskavitz J, Shea TB. Efficacy of a vitamin/nutriceutical formulation for moderate-stage to later-stage Alzheimer's disease: a placebo-controlled pilot study. *Am J Alzheimers Dis Other Demen*. 2009;**24**:27-33.

45. Aisen PS, Schneider LS, Sano M, et al. High-dose B vitamin supplementation and cognitive decline in Alzheimer disease: a randomized controlled trial. *JAMA*. 2008;**300**:1774-83.

46. Kessler H, Bayer TA, Bach D, et al. Intake of copper has no effect on cognition in patients with mild Alzheimer's disease: a pilot phase 2 clinical trial. *J Neural Transm*. 2008;**115**:1181-7.

47. Sun Y, Lu CJ, Chien KL, Chen ST, Chen RC. Efficacy of multivitamin supplementation containing vitamins B6 and B12 and folic acid as adjunctive treatment with a cholinesterase inhibitor in Alzheimer's disease: a 26-week, randomized, double-blind, placebo-controlled study in Taiwanese patients. *Clin Ther*. 2007;**29**:2204-14.

48. Connelly PJ, Prentice NP, Cousland G, Bonham J. A randomised double-blind placebo-controlled trial of folic acid supplementation of cholinesterase inhibitors in Alzheimer's disease. *Int J Geriatr Psychiatry*. 2008;**23**:155-60.

49. Demarin V, Podobnik SS, Storga-Tomic D, Kay G. Treatment of Alzheimer's disease with stabilized oral nicotinamide adenine dinucleotide: a randomized, double-blind study. *Drugs Exp Clin Res*. 2004;**30**:27-33.

50. Onofrj M, Thomas A, Luciano AL, et al. Donepezil versus vitamin E in Alzheimer's disease: Part 2: mild versus moderate-severe Alzheimer's disease. *Clin Neuropharmacol*. 2002;**25**:207-15.

51. Shah RC, Kamphuis PJ, Leurgans S, et al. The S-Connect study: Results from a randomized, controlled trial of Souvenaid in mild-to-moderate Alzheimer's disease. *Alzheimer's Research and Therapy*. 2013;**5**.

52. Scheltens P, Kamphuis PJGH, Verhey FRJ, et al. Efficacy of a medical food in mild Alzheimer's disease: A randomized, controlled trial. *Alzheimer's and Dementia*. 2010;**6**:1-10.e1.

53. Chan A, Paskavitz J, Remington R, Rasmussen S, Shea TB. Efficacy of a vitamin/nutriceutical formulation for early-stage Alzheimer's disease: A 1-year, open-label pilot study with an 16-month caregiver extension. *American Journal of Alzheimer's Disease and other Dementias*. 2009;**23**:571-85.

54. Henderson ST, Vogel JL, Barr LJ, Garvin F, Jones JJ, Costantini LC. Study of the ketogenic agent AC-1202 in mild to moderate Alzheimer's disease: A randomized, double-blind, placebo-controlled, multicenter trial. *Nutrition and Metabolism*. 2009;**6**.

55. Viswanathan A. High-dose B vitamin supplementation as a disease-modifying therapy in alzheimer disease. *Archives of Neurology*. 2009;**66**:520-2.

56. Anon. [Public title] S-Connect; [Scientific title] A randomized controlled trial to assess the efficacy of a medical food in patients with mild to moderate Alzheimer's disease using Alzheimer's disease medication. Netherlands Trial Register [wwwtrialregsiternl]; 2009.

57. Anon. A randomized, clinical trial of vitamin E and memantine in Alzheimer's disease (TEAM-AD). ClinicalTrialsgov [<http://clinicaltrialsgov];> 2007.

58. Blass JP. A nutritional brain metabolic enhancer for Alzheimer's disease. Clinical Trialsgov; 2002. p. 1-3.

59. Shinto LH. Fish Oil and Alpha Lipoic Acid in Mild Alzheimer's Disease. <http://wwwclinicaltrialsgov;> 2004.

60. Nct. A double blind placebo controlled randomized study to evaluate the efficacy and safety of bexarotene in patients with mild to moderate Alzheimer's disease. ClinicalTrialsgov [<http://clinicaltrialsgov];> 2013.

61. Pratt RD, Perdomo CA, Ieni JK. Long-term safety and tolerability of donepezil: results from a phase iii extension trial of patients with mild to moderately severe alzheimer's disease. European Journal of Neurology; 1999. p. 116.

62. Anon. [Public title] Souvenir II; [Scientific title] A randomized controlled trial to assess the efficacy of a food for special medical purposes (FSMP) in patients with mild Alzheimer's disease. Netherlands Trial Register (NTR) [wwwtrialregisternl/trialreg]; 2009.

63. Scheltens P, Kamphuis P. Medical nutrition in Alzheimer's disease: baseline characteristics of 3 Souvenaid clinical studies. European Journal of Pharmacology; 2011. p. e8-e9.

64. Henderson S. Safety, Tolerability and Efficacy Study of KetasynÂ? (AC-1202) Administered for Ninety Days in Subjects With Probable Alzheimer's Disease of Mild to Moderate Severity. ClinicalTrialsgov [<http://clinicaltrialsgov];> 2005.

65. Remington R, Bechtel C, Larsen D, et al. A Phase II Randomized Clinical Trial of a Nutritional Formulation for Cognition and Mood in Alzheimer's Disease. *J Alzheimers Dis*. 2015;**45**:395-405.

66. Akhondzadeh S, Noroozian M, Mohammadi M, Ohadinia S, Jamshidi AH, Khani M. Melissa officinalis extract in the treatment of patients with mild to moderate Alzheimer's disease: a double blind, randomised, placebo controlled trial. *J Neurol Neurosurg Psychiatry*. 2003;**74**:863-6.

67. Akhondzadeh S, Noroozian M, Mohammadi M, Ohadinia S, Jamshidi AH, Khani M. Salvia officinalis extract in the treatment of patients with mild to moderate Alzheimer's disease: a double blind, randomized and placebo-controlled trial. *J Clin Pharm Ther*. 2003;**28**:53-9.

68. Akhondzadeh S, Noroozian M, Mohammadi M, Ohadinia S, Moin M. Effects of a fixed dose of melissa officinalis extract in Alzheimer's disease: a randomized, placebo controlled trial. British Journal of Clinical Pharmacology; 2003. p. 443-4.

69. Schneider LS, DeKosky ST, Farlow MR, Tariot PN, Hoerr R, Kieser M. A randomized, double-blind, placebo-controlled trial of two doses of Ginkgo biloba extract in dementia of the Alzheimer's type. *Curr Alzheimer Res*. 2005;**2**:541-51.

70. Anon. [Public title] Saffron and Alzheimer; [Scientific title] Saffron extract in the treatment of mild to moderate Alzheimer’s disease: A double blind randomized controlled trial. Iranian Registry [wwwirctir]; 2006.

71. Vellas B, Andrieu S, Ousset PJ, Ouzid M, Mathiex-Fortunet H. The GuidAge study: Methodological issues A 5-year double-blind randomized trial of the efficacy of EGb 761(R) for prevention of Alzheimer disease in patients over 70 with a memory complaint. Neurology; 2006. p. S6-s11.

72. Wu YC, Zhao YB, Xie YY, Wang WZ. An open randomized comparative study of clinical efficacy and safety of huperzine A and rivastigmine in treatment of mild to moderate Alzheimer's disease. European Journal of Neurology; 2006. p. 198-9.

73. Department of Veterans Affairs. Development of NIC5-15 in the Treatment of Alzheimer's Disease, https://clinicaltrials.gov/ct2/show/NCT00470418. ClinicalTrialsgov [<http://clinicaltrialsgov];> 2007.

74. Baum L, Lam CWK, Cheung SKK, et al. Six-month randomized, placebo-controlled, double-blind, pilot clinical trial of curcumin in patients with Alzheimer disease [7]. *Journal of Clinical Psychopharmacology*. 2008;**28**:110-3.

75. Heo JH, Lee ST, Chu K, et al. An open-label trial of Korean red ginseng as an adjuvant treatment for cognitive impairment in patients with Alzheimer's disease. *Eur J Neurol*. 2008;**15**:865-8.

76. Lee ST, Chu K, Sim JY, Heo JH, Kim M. Panax ginseng enhances cognitive performance in Alzheimer disease. *Alzheimer Dis Assoc Disord*. 2008;**22**:222-6.

77. Anon. [Public title] Efficacy and safety of curcumin formulation in Alzheimer's disease; [Official/Scientific title] Phase II study of curcumin formulation (longvida) or placebo on plasma biomarkers and mental state in moderate to severe Alzheimer's disease or normal cognition. ClinicalTrialsgov [<http://clinicaltrialsgov];> 2009.

78. Akhondzadeh S, Sabet MS, Harirchian MH, et al. Saffron in the treatment of patients with mild to moderate Alzheimer's disease: a 16-week, randomized and placebo-controlled trial. *J Clin Pharm Ther*. 2010;**35**:581-8.

79. Akhondzadeh S, Shafiee Sabet M, Harirchian MH, et al. A 22-week, multicenter, randomized, double-blind controlled trial of Crocus sativus in the treatment of mild-to-moderate Alzheimer's disease. *Psychopharmacology (Berl)*. 2010;**207**:637-43.

80. Whanin Pharmaceutical Company. An Efficacy and Safety Study of INM-176 for the Treatment of Patients With Alzheimer Type Dementia, https://clinicaltrials.gov/ct2/show/NCT01245530. ClinicalTrialsgov [<http://clinicaltrialsgov];> 2010.

81. Rafii MS, Walsh S, Little JT, et al. A phase II trial of huperzine A in mild to moderate Alzheimer disease. *Neurology*. 2011;**76**:1389-94.

82. Heo JH, Lee ST, Chu K, et al. Heat-processed ginseng enhances the cognitive function in patients with moderately severe Alzheimer's disease. *Nutritional Neuroscience*. 2012;**15**:278-82.

83. James J. Peters Veterans Affairs Medical Center. A Single Site, Randomized, Double-blind, Placebo Controlled Trial of NIC5-15 in Subjects With Alzheimer's Disease, https://clinicaltrials.gov/ct2/show/NCT01928420. ClinicalTrialsgov [<http://clinicaltrialsgov];> 2012.

84. Ringman JM, Frautschy SA, Teng E, et al. Oral curcumin for Alzheimer's disease: tolerability and efficacy in a 24-week randomized, double blind, placebo-controlled study. *Alzheimers Res Ther*. 2012;**4**:43.

85. Nct. A randomized, double-blind, placebo-controlled, 6 month cross-over study to evaluate the efficacy of coconut oil (Fuel for Thoughtâ„¢) treatment for subjects with mild to moderate Alzheimer's disease. ClinicalTrialsgov [<http://clinicaltrialsgov];> 2013.

86. Farokhnia M, Shafiee Sabet M, Iranpour N, et al. Comparing the efficacy and safety of Crocus sativus L. with memantine in patients with moderate to severe Alzheimer's disease: a double-blind randomized clinical trial. *Hum Psychopharmacol*. 2014;**29**:351-9.

87. Chua KK, Wong A, Kwan PWL, et al. The efficacy and safety of the Chinese herbal medicine Di-Tan decoction for treating Alzheimer's disease: Protocol for a randomized controlled trial. *Trials*. 2015;**16**.

88. Pakdaman H, Harandi AA, Hatamian H, et al. Effectiveness and Safety of MLC601 in the Treatment of Mild to Moderate Alzheimer's Disease: A Multicenter, Randomized Controlled Trial. *Dement Geriatr Cogn Dis Extra*. 2015;**5**:96-106.

89. Winblad B, Andreasen N, Minthon L, et al. Safety, tolerability, and antibody response of active Abeta immunotherapy with CAD106 in patients with Alzheimer's disease: randomised, double-blind, placebo-controlled, first-in-human study. *Lancet Neurol*. 2012;**11**:597-604.

90. Grundman M, Capparelli E, Kim HT, et al. A multicenter, randomized, placebo controlled, multiple-dose, safety and pharmacokinetic study of AIT-082 (Neotrofin) in mild Alzheimer's disease patients. *Life Sci*. 2003;**73**:539-53.

91. Anon. [Public title] A study to evaluate the effects of MK0249 and an Alzheimer's disease medication on cognitive function in adults with Alzheimer's disease; [Scientific title] A randomized clinical trial to evaluate the single dose acute effects of MK0249 and donepezil on cognitive function in adult patients with Alzheimer's disease. ClinicalTrialsgov [<http://clinicaltrialsgov];> 2009.

92. Anon. Tolerability and safety of subcutaneous administration of affitope ad02 in mild to moderate Alzheimer's disease//randomized, controlled, parallel group, patient-blinded, single-center phase i pilot study to assess tolerability and safety of repeated sc administration of a single-dose of affitope ad02 applied with or without adjuvant to patients with mild to moderate Alzheimer's disease. ClinicalTrialsgov [<http://clinicaltrialsgov];> 2008.

93. Anon. [Public title] An escalating dose study to evaluate the safety, tolerability and pharmacokinetics of LNK 754 in elderly volunteers and in subjects with mild Alzheimer's disease; [Official/Scientific title] A double-blind, randomized, placebo-controlled, multiple, escalating dose study to evaluate the safety, tolerability and pharmacokinetics of LNK 754 in elderly volunteers and in subjects with mild Alzheimer's disease. ClinicalTrialsgov [<http://clinicaltrialsgov];> 2009.

94. Anon. A study of v950 in people with alzheimer disease or a double-blind, randomized, placebo-controlled, dose escalating study to evaluate the safety, tolerability, and immunogenicity of v950 formulated on aluminum-containing adjuvant with or without iscomatrixÂ? in patients with alzheimer disease. ClinicalTrialsgov [<http://clinicaltrialsgov];> 2007.

95. Anon. A clinical study to assess single and repeat doses of a new medication (GSK933776A) in patients with Alzheimer's disease Or A randomised, single-blind, placebo-controlled study to investigate the safety, tolerability, immunogenicity, pharmacokinetics and pharmacodynamics of intravenous infusion of GSK933776A in patients with Alzheimer's disease. ClinicalTrialsgov [<http://clinicaltrialsgov];> 2007.

96. Anon. A single blind, placebo-controlled, randomised study in mild to moderate Alzheimer's disease patients to assess the safety, tolerability, pharmacokinetics and pharmacodynamics of GSK239512, a selective histamine H3 receptor antagonist. ClinicalTrialsgov [<http://clinicaltrialsgov];> 2008.

97. Anon. Study evaluating single ascending doses of aab-001 vaccine sad japanese patients with alzheimers disease or a multicenter, randomized, double-blind, placebo-controlled, safety, tolerability, and pharmakokinetic study of single ascending doses of aab-001 in japanese patients with mild to moderate Alzheimer's disease. ClinicalTrialsgov [<http://clinicaltrialsgov];> 2006.

98. Nct. A multiple-dose study to evaluate the safety, tolerability, and pharmacokinetics of ABT-354 in subjects with mild-to-moderate Alzheimer's disease on stable doses of acetylcholinesterase inhibitors. ClinicalTrialsgov [<http://clinicaltrialsgov];> 2013.

99. Nct. Pilot Study: Lipoic Acid and Omega-3 Fatty Acid for Alzheimer's Disease Prevention. ClinicalTrialsgov [<http://clinicaltrialsgov];> 2013.

100. University of South Florida. Efficacy and Safety of Filgrastim in Alzheimer's Disease (FFAD), https://clinicaltrials.gov/ct2/show/NCT01617577. ClinicalTrialsgov [<http://clinicaltrialsgov];> 2011.

101. Nygaard HB, Wagner AF, Bowen GS, et al. A phase Ib multiple ascending dose study of the safety, tolerability, and central nervous system availability of AZD0530 (saracatinib) in Alzheimer's disease. *Alzheimers Res Ther*. 2015;**7**:35.

102. Sanofi. Activity of AVE1625 in Mild to Moderate Alzheimer's Patients, https://clinicaltrials.gov/ct2/show/NCT00380302. ClinicalTrialsgov [<http://clinicaltrialsgov>]

2006.

103. Lyketsos CG, Breitner JCS, Green RC, et al. Naproxen and celecoxib do not prevent AD in early results from a randomized controlled trial. *Neurology*. 2007;**68**:1800-8.

104. Tierney MC. Estrogen trial to delay the onset of Alzheimer's disease. <Http://wwwcontrolled-trialscom;> 2005.

105. Nct. The clinical study of pitavastatin treatment for group of mild to moderate Alzheimer's disease. ClinicalTrialsgov [<http://clinicaltrialsgov];> 2007.

106. Caramelli P, Laks J, Palmini AL, et al. Effects of galantamine and galantamine combined with nimodipine on cognitive speed and quality of life in mixed dementia: a 24-week, randomized, placebo-controlled exploratory trial (the REMIX study). *Arq Neuropsiquiatr*. 2014;**72**:411-7.

107. Winblad B, Giacobini E, Frolich L, et al. Phenserine efficacy in Alzheimer's disease. *J Alzheimers Dis*. 2010;**22**:1201-8.

108. Debattista C, Belanoff J. C-1073 (mifepristone) in the adjunctive treatment of Alzheimer's disease. *Current Alzheimer Research*. 2005;**2**:125-9.

109. Axonyx. Axonyx Announces Results Of Curtailed Phase III Clinical Trials For Phenserine In Alzheimer's Disease. Axonyxcom [<http://wwwaxonyxcom];> 2005.

110. Axonyx. Axonyx Reports Statistically Significant Result For Phenserine In Alzheimer's Disease Additional Analysis Results from Curtailed Phase III Clinical Trials. <http://wwwaxonyxcom/news_rsrs/indexhtml#;> 2005.

111. Leszek J, Inglot AD, Janusz M, et al. Colostrinin proline-rich polypeptide complex from ovine colostrum--a long-term study of its efficacy in Alzheimer's disease. Medical Science Monitor; 2002. p. Pi93-6.

112. Axonyx. Axonyx Announces That Phenserine Did Not Achieve Significant Efficacy In Phase III Alzheimer's Disease Trial. <http://wwwaxonyxcom/news_rsrs/indexhtml#;> 2005.

113. Moreno Moreno MDJ. Cognitive improvement in mild to moderate Alzheimer's dementia after treatment with the acetylcholine precursor choline alfoscerate: A multicenter, double-blind, randomized, placebo-controlled trial. *Clinical Therapeutics*. 2003;**25**:178-93.

114. Thal LJ, Calvani M, Amato A, Carta A. A 1-year controlled trial of acetyl-l-carnitine in early-onset AD. *Neurology*. 2000;**55**:805-10.

115. Barrett AM, Thal LJ. A 1 year controlled trial of acetyl-l-carnitine in early onset Alzheimer's disease. Neurology; 2001. p. 425.
