# Supplementary material for: Older patients are still under-represented in clinical trials of Alzheimer’s disease
Source: Alzheimers Res Ther. 2016 Aug 12;8:32. doi: 10.1186/s13195-016-0201-2 (PMC4982205; doi:10.1186/s13195-016-0201-2)
Supplement: Additional file 3: — List of studies included. (DOCX 34 kb) [file 13195_2016_201_MOESM3_ESM.docx]

**Additional file 3: list of included studies**

**References**

1. Saumier D, Duong A, Haine D, Garceau D, Sampalis J. Domain-specific cognitive effects of tramiprosate in patients with mild to moderate Alzheimer's disease: ADAS-cog subscale results from the Alphase Study. *J Nutr Health Aging*. 2009;**13**:808-12.

2. Aisen PS, Gauthier S, Ferris SH, et al. Tramiprosate in mild-to-moderate Alzheimer's disease - A randomized, double-blind, placebo-controlled, multi-centre study (the alphase study). *Archives of Medical Science*. 2011;**7**:102-11.

3. Vellas B. A Phase III study of the efficacy and safety of 3APS as add-on therapy in mild to moderate Alzheimer's disease. ClinicalTrialsgov [<http://clinicaltrialsgov];> 2005.

4. Bellus Health Inc. Evaluation of 3APS in Patients With Mild to Moderate Alzheimer’s Disease. https://clinicaltrials.gov/ct2/show/NCT00088673. ClinicalTrialsgov [<http://clinicaltrialsgov];> 2004.

5. Gilman S, Koller M, Black RS, et al. Clinical effects of Abeta immunization (AN1792) in patients with AD in an interrupted trial. *Neurology*. 2005;**64**:1553-62.

6. Shanghai Greenvalley Pharmaceutical Co. L. An Efficacy and Safety Study of Sodium Oligo-mannurarate (GV-971) Capsule for the Treatment of Alzheimer's Disease. https://[www.clinicaltrials.gov/ct2/show/NCT02293915?term=gv+971](http://www.clinicaltrials.gov/ct2/show/NCT02293915?term=gv+971). ClinicalTrialsgov [<http://clinicaltrialsgov];> 2014.

7. Shanghai Greenvalley Pharmaceutical Co. L. Safety, Efficacy and Dose Titration of Sodium Oligo-mannurarate Capsule on Mild to Moderate Alzheimer's Disease. https://clinicaltrials.gov/ct2/show/NCT01453569?term=NCT0145356. 2011.

8. Doody RS, Thomas RG, Farlow M, et al. Phase 3 trials of solanezumab for mild-to-moderate Alzheimer's disease. *N Engl J Med*. 2014;**370**:311-21.

9. Nct. Effect of passive immunization on the progression of mild Alzheimer's disease: solanezumab (LY2062430) versus placebo. ClinicalTrialsgov [<http://clinicaltrialsgov];> 2013.

10. Eli Lilly and Company. Effect of LY2062430 on the Progression of Alzheimer's Disease (EXPEDITION). https://clinicaltrials.gov/ct2/show/record/NCT00905372?term=EXPED. ClinicalTrialsgov [<http://clinicaltrialsgov];> 2009.

11. Company ELa. Effect of LY2062430 on the Progression of Alzheimer's Disease (EXPEDITION2). https://clinicaltrials.gov/ct2/show/NCT00904683?term=EXPEDITIO. ClinicalTrialsgov [<http://clinicaltrialsgov];> 2009.

12. Salloway S, Sperling R, Fox NC, et al. Two phase 3 trials of bapineuzumab in mild-to-moderate Alzheimer's disease. *N Engl J Med*. 2014;**370**:322-33.

13. Salloway S, Sperling R, Gilman S, et al. A phase 2 multiple ascending dose trial of bapineuzumab in mild to moderate Alzheimer disease. *Neurology*. 2009;**73**:2061-70.

14. Anon. [Public title] A long-term safety and tolerability study of bapineuzumab in Alzheimer disease patients; [Official/Scientific title] A phase 3 extension, multicenter, double-blind, long-term safety and tolerability trial of bapineuzumab (AAB-001, ELN115727) in subjects with Alzheimer disease who are apolipoprotein E e4 noncarriers and participated in study 3133K1-3000. ClinicalTrialsgov [<http://clinicaltrialsgov];> 2009.

15. Anon. Study evaluating the safety and efficacy of bapineuzumab in Alzheimer disease patients or a phase 3, multicenter, randomized, double-blind, placebo-controlled, parallel-group efficacy and safety trial of bapineuzumab in subjects with mild to moderate Alzheimer disease who are apolipoprotein e e4 carriers. ClinicalTrialsgov [<http://clinicaltrialsgov];> 2008.

16. Anon. [Public title] A long-term safety and tolerability extension study of bapineuzumab in Alzheimer disease patients; [Scientific title] A phase 3 extension, multicenter, long-term safety and tolerability trial of bapineuzumab (AAB-001, ELN115727) in subjects with Alzheimer disease who are apolipoprotein E e4 carriers and participated in study 3133K1-3001. ClinicalTrialsgov [<http://clinicaltrialsgov];> 2009.

17. Donoghue. A Multicenter, Randomized, Double Blind, Placebo Controlled, Multiple Ascending Dose, Safety, Tolerability, And Amyloid-Imaging Positron Emission Tomography (PET) Trial Of AAB 001 (ELN115727) In Patients With Mild To Moderate Alzheimer’s Disease (AD). ISRCTN Register; 2005.

18. Anon. A phase 3, multicenter, randomized, double-blind, placebo-controlled, parallel-group, efficacy and safety trial of bapineuzumab (aab-001, eln115727) in patients with mild to moderate Alzheimer's disease who are apolipoprotein e4 non- carriers. ClinicalTrialsgov [<http://clinicaltrialsgov];> 2007.

19. Pfizer. Study Evaluating the Safety and Efficacy of Bapineuzumab in Alzheimer Disease Patients. https://[www.clinicaltrials.gov/ct2/show/NCT00676143?term=Bapineu](http://www.clinicaltrials.gov/ct2/show/NCT00676143?term=Bapineu). ClinicalTrialsgov [<http://clinicaltrialsgov];> 2008.

20. Pfizer. Study Evaluating The Efficacy And Safety Of Bapineuzumab In Alzheimer Disease Patients. https://[www.clinicaltrials.gov/ct2/show/NCT00667810?term=Bapineu](http://www.clinicaltrials.gov/ct2/show/NCT00667810?term=Bapineu). ClinicalTrialsgov [<http://clinicaltrialsgov];> 2008.

21. Hoffmann-La Roche. A Study of Gantenerumab in Patients With Prodromal Alzheimer's Disease. https://clinicaltrials.gov/ct2/show/NCT01224106?term=NCT0122410. ClinicalTrialsgov [<http://clinicaltrialsgov];> 2010.

22. Hoffmann-La Roche. A Study of Gantenerumab in Patients With Mild Alzheimer Disease. https://clinicaltrials.gov/ct2/show/NCT02051608. ClinicalTrialsgov [<http://clinicaltrialsgov];> 2014.

23. Nct. A placebo-controlled, double-blind, parallel-group, Bayesian adaptive randomization design and dose regimen-finding study to evaluate safety, tolerability and efficacy of BAN2401 in subjects with early Alzheimer's disease. ClinicalTrialsgov [<http://clinicaltrialsgov];> 2013.

24. Genentech I. A Study to Evaluate the Efficacy and Safety of MABT5102A in Patients With Mild to Moderate Alzheimer's Disease (ABBY). https://clinicaltrials.gov/ct2/show/NCT01343966?term=NCT0134396. ClinicalTrialsgov [<http://clinicaltrialsgov];> 2011.

25. Biogen. 221AD301 Phase 3 Study of Aducanumab (BIIB037) in Early Alzheimer's Disease (ENGAGE). https://clinicaltrials.gov/ct2/show/NCT02477800?term=NCT0247780. ClinicalTrialsgov [<http://clinicaltrialsgov];> 2015.

26. Biogen. 221AD302 Phase 3 Study of Aducanumab (BIIB037) in Early Alzheimer's Disease (EMERGE). https://clinicaltrials.gov/ct2/show/NCT02484547?term=NCT0248454. ClinicalTrialsgov [<http://clinicaltrialsgov];> 2015.

27. Boada M, Ramos-Fernandez E, Guivernau B, et al. Treatment of Alzheimer disease using combination therapy with plasma exchange and haemapheresis with albumin and intravenous immunoglobulin: Rationale and treatment approach of the AMBAR (Alzheimer Management By Albumin Replacement) study. *Neurologia*. 2014.

28. Anon. [Public title] A phase 3 study evaluating safety and effectiveness of immune globulin intravenous (IGIV 10%) for the treatment of mild to moderate Alzheimer's disease; [Scientific title] A randomized, double-blind, placebo-controlled, two dose-arm, parallel study of the safety and effectiveness of immune globulin intravenous (Human), 10% (IGIV, 10%) for the treatment of mild to moderate Alzheimer's disease. ClinicalTrialsgov [<http://clinicaltrialsgov];> 2008.

29. Relkin NR. A Placebo-Controlled, Randomized, Double-Blind Phase II Clinical Study of Gammagard Intravenous Immunoglobulin (IVIg) for Treatment of Mild to Moderate Alzheimer's Disease. ClinicalTrialsgov [<http://clinicaltrialsgov];> 2006.

30. Baxalta US Inc. Phase 3 IGIV, 10% in Alzheimer´s Disease. https://clinicaltrials.gov/ct2/show/NCT01524887?term=NCT0152488. ClinicalTrialsgov [<http://clinicaltrialsgov];> 2012.

31. Ritchie CW, Bush AI, Mackinnon A, et al. Metal-protein attenuation with iodochlorhydroxyquin (clioquinol) targeting Abeta amyloid deposition and toxicity in Alzheimer disease: a pilot phase 2 clinical trial. *Arch Neurol*. 2003;**60**:1685-91.

32. Doody RS, Raman R, Farlow M, et al. A phase 3 trial of semagacestat for treatment of Alzheimer's disease. *N Engl J Med*. 2013;**369**:341-50.

33. Fleisher AS, Raman R, Siemers ER, et al. Phase 2 safety trial targeting amyloid beta production with a gamma-secretase inhibitor in Alzheimer disease. *Arch Neurol*. 2008;**65**:1031-8.

34. Siemers ER, Quinn JF, Kaye J, et al. Effects of a gamma-secretase inhibitor in a randomized study of patients with Alzheimer disease. *Neurology*. 2006;**66**:602-4.

35. Nct. Effects of ly450139, on the progression of Alzheimer's disease as compared with placebo or effect of ly450139 a y-secretase inhibitor, on the progression of Alzheimer's disease as compared with placebo. ClinicalTrialsgov [<http://clinicaltrialsgov];> 2008.

36. Anon. Effect of ?-secretase inhibition on the progression of Alzheimer's disease: ly450139 versus placebo. ClinicalTrialsgov [<http://clinicaltrialsgov];> 2008.

37. Green RC, Schneider LS, Amato DA, et al. Effect of tarenflurbil on cognitive decline and activities of daily living in patients with mild Alzheimer disease: a randomized controlled trial. *JAMA*. 2009;**302**:2557-64.

38. Wilcock GK, Black SE, Hendrix SB, Zavitz KH, Swabb EA, Laughlin MA. Efficacy and safety of tarenflurbil in mild to moderate Alzheimer's disease: a randomised phase II trial. *Lancet Neurol*. 2008;**7**:483-93.

39. Laughlin M. Phase 3 Multinational, Randomized, Double Blind, Placebo Controlled Study of the Effect of Daily Treatment With MPC-7869 on Measures of Cognition, Activities of Daily Living and Global Function in Subjects With Mild Dementia of the Alzheimer’s Type. ClinicalTrialsgov [<http://clinicaltrialsgov];> 2006.

40. Nct. A phase III, randomized, placebo-controlled, parallel-group, double-blind clinical trial to study the efficacy and safety of MK-8931 (SCH 900931) in subjects with amnestic mild cognitive impairment due to Alzheimer's disease (prodromal AD). ClinicalTrialsgov [<http://clinicaltrialsgov];> 2013.

41. Merck Sharp & Dohme Corp. Efficacy and Safety Trial of Verubecestat (MK-8931) in Participants With Prodromal Alzheimer's Disease (MK-8931-019) (APECS). https://clinicaltrials.gov/ct2/show/NCT01953601?term=NCT0195360. ClinicalTrialsgov [<http://clinicaltrialsgov];> 2013.

42. Eli Lilly and Company. An Efficacy and Safety Study of LY3314814 in Early Alzheimer's Disease (AMARANTH). https://clinicaltrials.gov/ct2/show/NCT02245737. ClinicalTrialsgov [<http://clinicaltrialsgov];> 2014.

43. Eisai Inc. Dose-Finding Study To Evaluate Safety, Tolerability, and Efficacy of E2609 in Subjects With Mild Cognitive Impairment Due to Alzheimer's Disease (Prodromal Alzheimer's Disease) and Mild to Moderate Dementia Due to Alzheimer's Disease. https://clinicaltrials.gov/ct2/show/NCT02322021?term=NCT0232202 ClinicalTrialsgov [<http://clinicaltrialsgov];> 2014.

44. Burstein AH, Grimes I, Galasko DR, Aisen PS, Sabbagh M, Mjalli AM. Effect of TTP488 in patients with mild to moderate Alzheimer's disease. *BMC Neurol*. 2014;**14**:12.

45. Burstein AH, Grimes I, Galasko DR, Aisen PS, Sabbagh M, Mjalli AMM. Effect of TTP488 in patients with mild to moderate Alzheimer's disease. *BMC Neurology*. 2014;**14**.

46. TransTech Pharma L. Evaluation of the Efficacy and Safety of Azeliragon (TTP488) in Patients With Mild Alzheimer's Disease (STEADFAST). https://clinicaltrials.gov/ct2/show/NCT02080364?term=NCT0208036. ClinicalTrialsgov [<http://clinicaltrialsgov];> 2014.

47. Lawlor B, Kennelly S, O'Dwyer S, et al. NILVAD protocol: a European multicentre double-blind placebo-controlled trial of nilvadipine in mild-to-moderate Alzheimer's disease. *BMJ Open*. 2014;**4**:e006364.

48. Sano M, Bell KL, Galasko D, et al. A randomized, double-blind, placebo-controlled trial of simvastatin to treat Alzheimer disease. *Neurology*. 2011;**77**:556-63.

49. Feldman HH, Doody RS, Kivipelto M, et al. Randomized controlled trial of atorvastatin in mild to moderate Alzheimer disease: LEADe. *Neurology*. 2010;**74**:956-64.

50. Jones RW, Kivipelto M, Feldman H, et al. The Atorvastatin/Donepezil in Alzheimer's Disease Study (LEADe): design and baseline characteristics. *Alzheimers Dement*. 2008;**4**:145-53.

51. Sparks DL, Connor DJ, Sabbagh MN, Petersen RB, Lopez J, Browne P. Circulating cholesterol levels, apolipoprotein E genotype and dementia severity influence the benefit of atorvastatin treatment in Alzheimer's disease: results of the Alzheimer's Disease Cholesterol-Lowering Treatment (ADCLT) trial. *Acta Neurol Scand Suppl*. 2006;**185**:3-7.

52. Sparks DL, Sabbagh MN, Connor DJ, et al. Atorvastatin for the treatment of mild to moderate Alzheimer disease: preliminary results. *Arch Neurol*. 2005;**62**:753-7.

53. Pfizer. An 80-week, randomized, multi-center, parallel-group, double-blind study of the efficacy and safety of atorvastatin 80 mg plus an acetylcholinesterase inhibitor versus an acetylcholinesterase inhibitor alone in the treatment of mild to moderate Alzheimer's disease. ClinicalTrialsgov [<http://clinicaltrialsgov];> 2005.

54. Wischik CM, Staff RT, Wischik DJ, et al. Tau aggregation inhibitor therapy: an exploratory phase 2 study in mild or moderate Alzheimer's disease. *J Alzheimers Dis*. 2015;**44**:705-20.

55. TauRx Therapeutics Ltd. Safety and Efficacy Study Evaluating TRx0237 in Subjects With Mild to Moderate Alzheimer's Disease. https://[www.clinicaltrials.gov/ct2/show/NCT01689246](http://www.clinicaltrials.gov/ct2/show/NCT01689246). ClinicalTrialsgov [<http://clinicaltrialsgov];> 2012.

56. TauRx Therapeutics Ltd. Safety and Efficacy Study Evaluating TRx0237 in Subjects With Mild Alzheimer's Disease. https://[www.clinicaltrials.gov/ct2/show/NCT01689233](http://www.clinicaltrials.gov/ct2/show/NCT01689233). ClinicalTrialsgov [<http://clinicaltrialsgov];> 2012.

57. TauRx Therapeutics Ltd. TRx0014 in Patients With Mild or Moderate Alzheimer's Disease. https://clinicaltrials.gov/show/NCT00515333. 2007.

58. Lovestone S, Boada M, Dubois B, et al. A phase II trial of tideglusib in Alzheimer's disease. *J Alzheimers Dis*. 2015;**45**:75-88.

59. Noscira SA. Efficacy, Safety and Tolerability of Tideglusib to Treat Mild-to-Moderate Alzheimer's Disease Patients (ARGO). https://clinicaltrials.gov/ct2/show/NCT01350362?term=NCT0135036. ClinicalTrialsgov [<http://clinicaltrialsgov];> 2011.

60. Claxton A, Baker LD, Hanson A, et al. Long-Acting Intranasal Insulin Detemir Improves Cognition for Adults with Mild Cognitive Impairment or Early-Stage Alzheimer's Disease Dementia. *J Alzheimers Dis*. 2014.

61. Craft S, Baker LD, Montine TJ, et al. Intranasal insulin therapy for Alzheimer disease and amnestic mild cognitive impairment: a pilot clinical trial. *Arch Neurol*. 2012;**69**:29-38.

62. Nct. Therapeutic effects of intranasally-administered insulin in adults with amnestic mild cognitiveimpairment (aMCI) or mild Alzheimer's disease (AD). ClinicalTrialsgov [<http://clinicaltrialsgov];> 2013.

63. Rosenbloom MH, Barclay TR, Pyle M, et al. A single-dose pilot trial of intranasal rapid-acting insulin in apolipoprotein E4 carriers with mild-moderate Alzheimer's disease. *CNS Drugs*. 2014;**28**:1185-9.

64. Wake Forest School of Medicine. Study of Nasal Insulin to Fight Forgetfulness - Long-acting Insulin Detemir - 120 Days (SL120). https://clinicaltrials.gov/ct2/show/study/NCT01595646. ClinicalTrialsgov [<http://clinicaltrialsgov];> 2012.

65. HealthPartners Institute for Education and Research. Safety and Effectiveness Study of Intranasal Insulin Glulisine on Cognitive and Memory in Mild-Mod AD Patients. https://clinicaltrials.gov/ct2/show/NCT01436045. ClinicalTrialsgov [<http://clinicaltrialsgov];> 2011.

66. Harrington C, Sawchak S, Chiang C, et al. Rosiglitazone does not improve cognition or global function when used as adjunctive therapy to AChE inhibitors in mild-to-moderate Alzheimer's disease: two phase 3 studies. *Curr Alzheimer Res*. 2011;**8**:592-606.

67. Gold M, Alderton C, Zvartau-Hind M, et al. Rosiglitazone monotherapy in mild-to-moderate Alzheimer's disease: results from a randomized, double-blind, placebo-controlled phase III study. *Dement Geriatr Cogn Disord*. 2010;**30**:131-46.

68. Watson GS, Reger MA, Cholerton BA, et al. Rosiglitazone Preserves Cognitive Functions in Patients with Early Alzheimer's Disease. Neurobiology of Aging; 2004. p. 83.

69. Nct. A 54-week, double-blind, randomized, placebo-controlled, parallel-group study to investigate the effects of rosiglitazone (Extended Release Tablets) as adjunctive therapy to donepezil on cognition and overall clinical response in APOE e4-stratified subjects with mild to moderate Alzheimer's disease (REFLECT-2). ClinicalTrialsgov [<http://clinicaltrialsgov];> 2006.

70. GlaxoSmithKline. A 54-week, double-blind, randomized, placebo-controlled, parallel-group study to investigate the effects of rosiglitazone (Extended Release Tablets) as adjunctive therapy to acetylcholinesterase inhibitors on cognition and overall clinical response in APOE e4-stratified subjects with mild to moderate Alzheimer's disease (REFLECT-3). ClinicalTrialsgov [<http://clinicaltrialsgov];> 2006.

71. Sato T, Hanyu H, Hirao K, Kanetaka H, Sakurai H, Iwamoto T. Efficacy of PPAR-gamma agonist pioglitazone in mild Alzheimer disease. *Neurobiol Aging*. 2011;**32**:1626-33.

72. Hanyu H, Sato T, Kiuchi A, Sakurai H, Iwamoto T. Pioglitazone improved cognition in a pilot study on patients with Alzheimer's disease and mild cognitive impairment with diabetes mellitus. *J Am Geriatr Soc*. 2009;**57**:177-9.

73. Accera Inc. AC-1204 Long-term Efficacy Response Trial (ALERT Protocol). https://clinicaltrials.gov/ct2/show/NCT01211782. ClinicalTrialsgov [<http://clinicaltrialsgov];> 2010.

74. Accera Inc. AC-1204 26-Week Long Term Efficacy Response Trial With Optional Open-label Ext (NOURISH AD). https://clinicaltrials.gov/ct2/show/NCT01741194. ClinicalTrialsgov [<http://clinicaltrialsgov];> 2012.

75. Schwam EM, Nicholas T, Chew R, et al. A multicenter, double-blind, placebo-controlled trial of the PDE9A inhibitor, PF-04447943, in Alzheimer's disease. *Curr Alzheimer Res*. 2014;**11**:413-21.

76. Anon. [Public title] A study of PF-04447943 compared to placebo in subjects with mild to moderate Alzheimer's disease; [Scientific title] A phase 2 multicenter, double-blind, placebo-controlled, parallel group study of PF-04447943 in subjects with mild to moderate Alzheimer's disease. ClinicalTrialsgov [<http://clinicaltrialsgov];> 2009.

77. Boehringer Ingelheim. Alzheimer Disease Proof of Concept Study With BI 409306 Versus Placebo. https://clinicaltrials.gov/ct2/show/NCT02240693?term=NCT0224069. ClinicalTrialsgov [<http://clinicaltrialsgov];> 2014.

78. Boehringer Ingelheim. BI 409306 in Patients With Cognitive Impairment Due to Alzheimer's Disease. https://clinicaltrials.gov/ct2/show/NCT02337907?term=NCT0233790. ClinicalTrialsgov [<http://clinicaltrialsgov];> 2014.

79. Nct. Efficacy of Zydena (Udenafil) on cognitive function of Alzheimer's disease patients: a randomized, double blind, placebo-controlled multicenter study. ClinicalTrialsgov [<http://clinicaltrialsgov];> 2013.

80. Merck. MK-0952 in Patients With Mild-to-Moderate Alzheimer's Disease. ClinicalTrialsgov [<http://clinicaltrialsgov];> 2006.

81. Grove RA, Harrington CM, Mahler A, et al. A randomized, double-blind, placebo-controlled, 16-week study of the H3 receptor antagonist, GSK239512 as a monotherapy in subjects with mild-to-moderate Alzheimer's disease. *Curr Alzheimer Res*. 2014;**11**:47-58.

82. Anon. [Public title] Study to evaluate the efficacy and safety of GSK239512 in Alzheimer's disease; [Official/Scientific title] A randomised, double-blind, placebo-controlled study to evaluate the efficacy and safety of the H3 receptor antagonist, GSK239512 in subjects with mild to moderate Alzheimer's disease. ClinicalTrialsgov [<http://clinicaltrialsgov];> 2009.

83. Egan M, Yaari R, Liu L, et al. Pilot randomized controlled study of a histamine receptor inverse agonist in the symptomatic treatment of AD. *Curr Alzheimer Res*. 2012;**9**:481-90.

84. Haig GM, Pritchett Y, Meier A, et al. A randomized study of H3 antagonist ABT-288 in mild-to-moderate Alzheimer's dementia. *Journal of Alzheimer's Disease*. 2014;**42**:959-71.

85. Anon. Efficacy and safety study of ABT-288 in subjects with mild-to-moderate Alzheimer's disease. ClinicalTrialsgov [<http://clinicaltrialsgov];> 2009.

86. Carlson MC, Tschanz JT, Norton MC, Welsh-Bohmer K, Martin BK, Breitner JC. H2 histamine receptor blockade in the treatment of Alzheimer disease: a randomized, double-blind, placebo-controlled trial of nizatidine. *Alzheimer Dis Assoc Disord*. 2002;**16**:24-30.

87. Sanofi. Effect of Different Doses of SAR110894D on Cognition in Patients With Mild to Moderate Alzheimer's Disease on Donepezil. https://clinicaltrials.gov/ct2/show/NCT01266525. ClinicalTrialsgov [<http://clinicaltrialsgov];> 2010.

88. Anon. Safety and efficacy of S 38093 versus placebo in patients with Alzheimer’s Disease. An international, multi-centre, randomised, double-blind, placebo-controlled phase IIa study. UK Clinical Research Network [wwwukcrnorguk]; 2009.

89. UK Clinical Research Network Study Portfolio. Efficacy and safety of 3 doses of S 38093 (2, 5 and 20 mg/day) in co-administration with Donepezil (10 mg/day) in patients with moderate Alzheimer’s Disease. A 24-week international, multi-centre, randomised, double-blind, placebo-controlled phase IIb study. <http://public.ukcrn.org.uk/search/StudyDetail.aspx?StudyID=11864>.

90. Bedwell E. A study of the safety and efficacy of multiple doses of ABT-089 in subjects with Alzheimer's disease. ClinicalTrialsgov [<http://clinicaltrialsgov];> 2003.

91. Lenz RA, Pritchett YL, Berry SM, et al. Adaptive, Dose-finding Phase 2 Trial Evaluating the Safety and Efficacy of ABT-089 in Mild to Moderate Alzheimer Disease. *Alzheimer Dis Assoc Disord*. 2015.

92. Anon. [Public title] A study of RO5313534 as add-on to donepezil treatment in patients with mild to moderate Alzheimer's disease; [Scientific title] A dose-ranging, randomized, double-blind , placebo-controlled study of the effect of RO5313534, used as add-on therapy to donepezil, on cognitive function in patients with mild to moderate symptoms of Alzheimer's disease. ClinicalTrialsgov [<http://clinicaltrialsgov];> 2009.

93. Anon. A dose ranging, randomised, double blind, parallel group placebo-controlled multi-centre study of RO5313534 used as an add-on to donepezil treatment in patients with mild to moderate symptoms of Alzheimer’s Disease. UK Clinical Research Network [wwwukcrnorguk]; 2009.

94. Mitsubishi Tanabe Pharma Corporation. Safety and Efficacy of MT-4666. https://clinicaltrials.gov/ct2/show/NCT01764243?term=NCT0176424 ClinicalTrialsgov [<http://clinicaltrialsgov];> 2012.

95. FORUM Pharmaceuticals Inc. Study of the Safety and Effectiveness of Two Doses of Investigational Study Drug EVP-6124 in Subjects With Alzheimer's Disease. https://clinicaltrials.gov/ct2/show/NCT01969136?term=EVP-6124-0. ClinicalTrialsgov [<http://clinicaltrialsgov];> 2013.

96. FORUM Pharmaceuticals Inc. Study of the Safety and Effectiveness of Two Doses of Investigational Study Drug EVP-6124 in Subjects With Alzheimer's Disease. https://clinicaltrials.gov/ct2/show/NCT01969123. ClinicalTrialsgov [<http://clinicaltrialsgov];> 2013.

97. Kim SY, Choi SH, Rollema H, et al. Phase II crossover trial of varenicline in mild-to-moderate Alzheimer's disease. *Dement Geriatr Cogn Disord*. 2014;**37**:232-45.

98. Anon. [Public title] Evaluation of the efficacy of varenicline on cognition, safety, tolerability and pharmacokinetics in subjects with mild-to-moderate Alzheimer's disease; [Official title] A phase 2 multicenter, double-blind, placebo-controlled, crossover trial of varenicline tartrate (CP-526,555) in patients with mild to moderate Alzheimer's disease. ClinicalTrialsgov [<http://clinicaltrialsgov];> 2009.

99. Frolich L, Ashwood T, Nilsson J, Eckerwall G. Effects of AZD3480 on cognition in patients with mild-to-moderate alzheimer's disease: A phase IIb dose-finding study. *Journal of Alzheimer's Disease*. 2011;**24**:363-74.

100. Targacept Inc. Efficacy, Safety, & Tolerability of AZD3480 Patients With Mild to Moderate Dementia of the Alzheimer's Type (AD). https://clinicaltrials.gov/ct2/show/NCT01466088?term=NCT0146608. ClinicalTrialsgov [<http://clinicaltrialsgov];> 2011.

101. Novartis Pharmaceuticals C. Effect of AQW051 in Patients With Memory Impairment. https://clinicaltrials.gov/ct2/show/NCT00582855?term=NCT00582855&rank=1. ClinicalTrialsgov [<http://clinicaltrialsgov];> 2007.

102. Anon. A randomized, double-blind, placebo and active-controlled, parallel group study to evaluate the efficacy and safety of ABT-126 in subjects with mild to moderate Alzheimer's disease. UK Clinical Research Network [<http://publicukcrnorguk];> 2009.

103. AbbVie. Evaluate the Efficacy and Safety of ABT-126 in Subjects With Mild to Moderate Alzheimer's Disease on Stable Doses of Acetylcholinesterase Inhibitors. ClinicalTrialsgov [<http://clinicaltrialsgov];> 2012.

104. AbbVie. Evaluate the Efficacy and Safety of ABT-126 in Subjects With Mild to Moderate Alzheimer's Disease. https://clinicaltrials.gov/ct2/show/NCT01527916. ClinicalTrialsgov [<http://clinicaltrialsgov];> 2012.

105. Nct. A seamless phase IIa/IIb, multicenter, randomized, double-blind, placebo-controlled, parallel group trial to evaluate the efficacy and safety of MK-7622 as an adjunctive therapy to donepezil for symptomatic treatment in subjects with Alzheimer's disease. ClinicalTrialsgov [<http://clinicaltrialsgov];> 2013.

106. Thal LJ, Forrest M, Loft H, Mengel H. Lu 25-109, a muscarinic agonist, fails to improve cognition in Alzheimer's disease. Lu25-109 Study Group. *Neurology*. 2000;**54**:421-6.

107. Boehringer Ingelheim. Efficacy and Safety of Talsaclidine (Free Base) in Patients With Mild to Moderate Dementia of Alzheimer Type. https://clinicaltrials.gov/ct2/show/NCT02249351?term=NCT0224935. ClinicalTrialsgov [<http://clinicaltrialsgov];> 2014.

108. Boehringer Ingelheim. Efficacy and Safety of Talsaclidine in Patients With Mild to Moderate Dementia of Alzheimer Type. https://clinicaltrials.gov/ct2/show/NCT02249403?term=NCT0224940. ClinicalTrialsgov [<http://clinicaltrialsgov];> 2014.

109. Maher-Edwards G, Dixon R, Hunter J, et al. SB-742457 and donepezil in Alzheimer disease: a randomized, placebo-controlled study. *Int J Geriatr Psychiatry*. 2011;**26**:536-44.

110. Maher-Edwards G, Zvartau-Hind M, Hunter AJ, et al. Double-blind, controlled phase II study of a 5-HT6 receptor antagonist, SB-742457, in Alzheimer's disease. *Curr Alzheimer Res*. 2010;**7**:374-85.

111. Anon. Study az3110866, a fixed dose study of sb-742457 versus placebo when added to existing donepezil treatment in subjects with mild-to-moderate Alzheimer's disease. ClinicalTrialsgov [<http://clinicaltrialsgov];> 2008.

112. GlaxoSmithKline. A phase IIa/b double-blind, randomised, placebo-controlled, linear trend design dose-ranging study to investigate the effects of 24 weeks of monotherapy with SB-742457 on cognition in subjects with mild to moderate Alzheimer's disease. ClinicalTrialsgov [<http://clinicaltrialsgov];> 2005.

113. GlaxoSmithKline. A Double-Blind, Randomised, Placebo-Controlled, Parallel Group Study to Investigate the Effects of SB-742457, Donepezil and Placebo on Cognition in Subjects With Mild to Moderate Alzheimer's Disease. ClinicalTrialsgov [wwwclinicaltrialsgov]; 2006.

114. GlaxoSmithKline. Study of SB-742457 or Donepezil Versus Placebo in Subjects With Mild-to-moderate Alzheimer's Disease. https://clinicaltrials.gov/ct2/show/NCT00708552?term=NCT0070855. ClinicalTrialsgov [<http://clinicaltrialsgov];> 2008.

115. Wilkinson D, Windfeld K, Colding-Jorgensen E. Safety and efficacy of idalopirdine, a 5-HT6 receptor antagonist, in patients with moderate Alzheimer's disease (LADDER): A randomised, double-blind, placebo-controlled phase 2 trial. *The Lancet Neurology*. 2014;**13**:1092-9.

116. Anon. [Public title] Lu AE58054 added to donepezil for the treatment for moderate Alzheimer's disease; [Official/Scientific title] Randomised, double-blind, parallel-group, placebo-controlled, fixed-dose study of Lu AE58054 in patients with moderate Alzheimer's disease treated with donepezil. ClinicalTrialsgov [<http://clinicaltrialsgov];> 2009.

117. Nct. Randomised, double-blind, parallel-group, placebo-controlled, fixed-dose study of Lu AE58054 in patients with mild - moderate Alzheimer's disease treated with donepezil. ClinicalTrialsgov [<http://clinicaltrialsgov];> 2013.

118. H. Lundbeck A/S. Lu AE58054 in Patients With Mild to Moderate Alzheimer's Disease Treated With an Acetylcholinesterase Inhibitor (STARBRIGHT). https://clinicaltrials.gov/ct2/show/NCT02006654?term=NCT0200665. ClinicalTrialsgov [<http://clinicaltrialsgov];> 2013.

119. Pfizer. Study Comparing 3 Dosage Levels Of SAM-531 In Outpatients With Mild To Moderate Alzheimer Disease. https://clinicaltrials.gov/ct2/show/NCT00895895?term=NCT0089589. ClinicalTrialsgov [<http://clinicaltrialsgov];> 2012.

120. Anon. A Randomized, Multicenter, Double-Blind, Placebo-Controlled, 18-Month Study of the Efficacy of SR57746A in Patients with Mild-to-Moderate Dementia of the Alzheimer’s Type. <http://wwwcontrolled-trialscom/mrct;> 2005.

121. Sanofi. Study of Xaliproden (SR57746A) in Patients With Mild-to-Moderate Dementia of the Alzheimer's Type. https://clinicaltrials.gov/ct2/show/NCT00104013?term=Xaliproden&r. ClinicalTrialsgov [<http://clinicaltrialsgov];> 2005.

122. Sanofi. Study of the Effect of SR57667B in Patients With Alzheimer's Disease. https://clinicaltrials.gov/ct2/show/NCT00285025?term=NCT0028502. ClinicalTrialsgov [<http://clinicaltrialsgov];> 2006.

123. A 3-Month, Randomized, Double-Blind, Placebo-Controlled, Multicenter, Safety, Tolerability, and Efficacy Study Of 3 Doses Of Lecozotan (SRA-333) SR In Outpatients With Mild To Moderate Alzheimer's Disease With Donepezil As Active Control. ClinicalTrialsgov [<http://clinicaltrialsgov];> 2005.

124. Wyeth Study Evaluating the Safety, Tolerability, and Efficacy of Lecozotan SR in Outpatients With Alzheimer's Disease. https://clinicaltrials.gov/ct2/show/NCT00277810?term=NCT0027781. ClinicalTrialsgov [<http://clinicaltrialsgov];> 2006.

125. Anon. Study of prx-03140 monotherapy in subjects with Alzheimer's disease or a phase 2, multicenter, randomized, double-blind, placebo-controlled, parallel group study to evaluate the efficacy and safety of prx-03140 as monotherapy in subjects with Alzheimer's disease. ClinicalTrialsgov [<http://clinicaltrialsgov];> 2008.

126. Epix Pharmaceuticals I. A Study of PRX-03140 in Subjects With Alzheimer's Disease Receiving a Stable Dose of Donepezil. https://clinicaltrials.gov/ct2/show/NCT00672945?term=NCT0067294. ClinicalTrialsgov [<http://clinicaltrialsgov];> 2008.

127. Sunovion. Safety and Efficacy Study of AC-3933 in Adults With Mild to Moderate Alzheimer's Disease. https://clinicaltrials.gov/ct2/show/results/NCT00359944?sect=X01256. ClinicalTrialsgov [<http://clinicaltrialsgov];> 2006.

128. Mohs RC, Shiovitz TM, Tariot PN, Porsteinsson AP, Baker KD, Feldman PD. Atomoxetine augmentation of cholinesterase inhibitor therapy in patients with Alzheimer disease: 6-month, randomized, double-blind, placebo-controlled, parallel-trial study. *Am J Geriatr Psychiatry*. 2009;**17**:752-9.

129. Eli L, Company. Atomoxetine augmentation of cholinesterase inhibitor therapy in patients with Alzheimer's disease. ClinicalTrialsgov [<http://clinicaltrialsgov];> 2005.

130. Anon. A randomized, double-blind, placebo-controlled evalution of the safety and efficacy of neramexane monotherapy in patients with moderate to severe dementia of the Alzheimer's type. ClinicalTrialsgov [<http://clinicaltrialsgov];> 2004.

131. Boehringer I. A phase II double-blind, randomized, placebo-controlled, multicenter, safety and efficacy evaluation of three doses of NS 2330 in patients with probable mild to moderate Alzheimer's disease. ClinicalTrialsgov [<http://clinicaltrialsgov];> 2005.

132. Orion Corporation. Safety and Efficacy of ORM-12741 in Patients With Alzheimer's Disease (ALPO). https://clinicaltrials.gov/ct2/show/NCT01324518?term=NCT0132451. 2011.

133. Teva Pharmaceutical Industries. Rasagiline 1 mg and 2 mg Added to Aricept 10 mg Daily in Patients With Mild to Moderate Alzheimer's Disease (AD). https://clinicaltrials.gov/ct2/show/NCT00104273?term=NCT0010427. ClinicalTrialsgov [<http://clinicaltrialsgov];> 2005.

134. Saegis Pharmaceuticals. SGS742 in Patients With Mild to Moderate Alzheimer's Disease (AD). https://clinicaltrials.gov/ct2/show/NCT00093951?term=NCT0009395. ClinicalTrialsgov [<http://clinicaltrialsgov];> 2004.

135. Eli Lilly and Company. Efficacy and Safety of LY451395 in Patients With Probable Alzheimer's Disease. https://clinicaltrials.gov/ct2/show/NCT00051909?term=NCT0005190. ClinicalTrialsgov [<http://clinicaltrialsgov];> 2003.

136. Bentham P, Gray R, Sellwood E, Hills R, Crome P, Raftery J. Aspirin in Alzheimer's disease (AD2000): a randomised open-label trial. *Lancet Neurol*. 2008;**7**:41-9.

137. de Jong D, Jansen R, Hoefnagels W, et al. No effect of one-year treatment with indomethacin on Alzheimer's disease progression: a randomized controlled trial. *PLoS One*. 2008;**3**:e1475.

138. Pasqualetti P, Bonomini C, Dal Forno G, et al. A randomized controlled study on effects of ibuprofen on cognitive progression of Alzheimer's disease. *Aging Clin Exp Res*. 2009;**21**:102-10.

139. Aisen PS, Schmeidler J, Pasinetti GM. Randomized pilot study of nimesulide treatment in Alzheimer's disease. *Neurology*. 2002;**58**:1050-4.

140. Reines SA, Block GA, Morris JC, et al. Rofecoxib: no effect on Alzheimer's disease in a 1-year, randomized, blinded, controlled study. *Neurology*. 2004;**62**:66-71.

141. Aisen PS, Schafer KA, Grundman M, et al. Effects of rofecoxib or naproxen vs placebo on Alzheimer disease progression: a randomized controlled trial. *JAMA*. 2003;**289**:2819-26.

142. Beck K. The safety and efficacy of an Investigational Drug (VIOXX) in Delaying the progression of Alzheimer's Disease. ClinicalTrialsgov [<http://clinicaltrialsgov];> 2000.

143. Soininen H, West C, Robbins J, Niculescu L. Long-term efficacy and safety of celecoxib in Alzheimer's disease. *Dement Geriatr Cogn Disord*. 2007;**23**:8-21.

144. JSW Lifesciences. Efficacy and Safety of Lornoxicam in Patients With Mild to Moderate Probable Alzheimer´s Disease. https://clinicaltrials.gov/ct2/show/NCT01117948. 2010.

145. Van Gool WA, Weinstein HC, Scheltens P, Walstra GJ. Effect of hydroxychloroquine on progression of dementia in early Alzheimer's disease: an 18-month randomised, double-blind, placebo-controlled study. *Lancet*. 2001;**358**:455-60.

146. Due BR. Effects of ONO-2506PO in Patients with Alzheimer's Disease. <http://wwwclinicaltrialsgov/;> 2004.

147. Jacoby R. Phase II randomised, double blind, placebo controlled, parallel group study to investigate the safety, effect on cognition and pharmacokinetic profile of PYM50028 in subjects with mild dementia of the Alzheimer's type. ClinicalTrialsgov [<http://clinicaltrialsgov];> 2005.

148. Belanoff JK, Jurik J, Schatzberg LD, DeBattista C, Schatzberg AF. Slowing the progression of cognitive decline in Alzheimer's disease using mifepristone. *J Mol Neurosci*. 2002;**19**:201-6.

149. Pomara N, Doraiswamy PM, Tun H, Ferris S. Mifepristone (RU 486) for Alzheimer's disease. *Neurology*. 2002;**58**:1436.

150. Shaw Y. A double-blind, placebo-controlled trial of the safety and efficacy of C-1073 (Mifepristone) as adjunctive therapy in Alzheimer's disease. ClinicalTrialsgov [<http://clinicaltrialsgov];> 2005.

151. Aisen PS, Davis KL, Berg JD, et al. A randomized controlled trial of prednisone in Alzheimer's disease. Alzheimer's Disease Cooperative Study. *Neurology*. 2000;**54**:588-93.

152. Marek GJ, Katz DA, Meier A, et al. Efficacy and safety evaluation of HSD-1 inhibitor ABT-384 in Alzheimer's disease. *Alzheimers Dement*. 2014;**10**:S364-73.

153. Bowen RL, Perry G, Xiong C, Smith MA, Atwood CS. A Clinical Study of Lupron Depot in the Treatment of Women with Alzheimer's Disease: Preservation of Cognitive Function in Patients Taking an Acetylcholinesterase Inhibitor and Treated with High Dose Lupron Over 48 Weeks. *J Alzheimers Dis*. 2014.

154. Powers C. A Double-Blind Placebo-Controlled Study of VP4896 For the Treatment of Mild-to-Moderate Alzheimer’s Disease. ClinicalTrialsgov [<http://clinicaltrialsgov];> 2005.

155. Voyager Pharmaceutical C. Leuprolide Acetate Stabilizes Cognitive and Functional Decline in Women With Alzheimer's Disease, Study Shows. wwwvoyagerpharmacom; 2006.

156. Henderson. Raloxifene for Women With Alzheimer's Disease Or Raloxifene in Women With AD: Randomized Controlled Trial. ClinicalTrialsgov [<http://clinicaltrialsgov];> 2006.

157. Wharton W, Baker LD, Gleason CE, et al. Short-term hormone therapy with transdermal estradiol improves cognition for postmenopausal women with Alzheimer's disease: results of a randomized controlled trial. *J Alzheimers Dis*. 2011;**26**:495-505.

158. Yoon BK, Kim DK, Kang Y, Kim JW, Shin MH, Na DL. Hormone replacement therapy in postmenopausal women with Alzheimer's disease: a randomized, prospective study. *Fertil Steril*. 2003;**79**:274-80.

159. Wang PN, Liao SQ, Liu RS, et al. Effects of estrogen on cognition, mood, and cerebral blood flow in AD: a controlled study. *Neurology*. 2000;**54**:2061-6.

160. Mulnard RA, Cotman CW, Kawas C, et al. Estrogen replacement therapy for treatment of mild to moderate Alzheimer disease: a randomized controlled trial. Alzheimer's Disease Cooperative Study. *JAMA*. 2000;**283**:1007-15.

161. Henderson VW, Paganini-Hill A, Miller BL, et al. Estrogen for Alzheimer's disease in women: randomized, double-blind, placebo-controlled trial. *Neurology*. 2000;**54**:295-301.

162. Wolkowitz OM, Kramer JH, Reus VI, et al. DHEA treatment of Alzheimer's disease: a randomized, double-blind, placebo-controlled study. *Neurology*. 2003;**60**:1071-6.

163. Lu PH, Masterman DA, Mulnard R, et al. Effects of testosterone on cognition and mood in male patients with mild Alzheimer disease and healthy elderly men. *Archives of Neurology*. 2006;**63**:177-85.

164. Sevigny JJ, Ryan JM, van Dyck CH, Peng Y, Lines CR, Nessly ML. Growth hormone secretagogue MK-677: no clinical effect on AD progression in a randomized trial. *Neurology*. 2008;**71**:1702-8.

165. Piette F, Belmin J, Vincent H, et al. Masitinib as an adjunct therapy for mild-to-moderate Alzheimer's disease: A randomised, placebo-controlled phase 2 trial. *Alzheimer's Research and Therapy*. 2011;**3**.

166. Anon. [Public title] Activity of masitinib (AB1010) in mild to moderate Alzheimer's disease; [Scientific title] A multicenter, double-blind, placebo-controlled, randomized, parallel-group study to evaluate the efficacy of oral AB1010 in adults patients with mild to moderate Alzheimer-type disease. ClinicalTrialsgov [<http://clinicaltrialsgov];> 2009.

167. Nct. A multicenter, double-blind, placebo-controlled, randomised, parallel-group phase 3 study to evaluate the safety and efficacy of masitinib in patients with mild to moderate Alzheimer's disease. ClinicalTrialsgov [<http://clinicaltrialsgov];> 2013.

168. Doody RS, Gavrilova SI, Sano M, et al. Effect of dimebon on cognition, activities of daily living, behaviour, and global function in patients with mild-to-moderate Alzheimer's disease: a randomised, double-blind, placebo-controlled study. *Lancet*. 2008;**372**:207-15.

169. Sweetlove M. Phase III CONCERT trial of latrepirdine: Negative results. *Pharmaceutical Medicine*. 2012;**26**:113-5.

170. Anon. [Public title] A phase 3 efficacy study of dimebon in patients with moderate to severe Alzheimer's disease; [Scientific title] A phase 3, multi-center, randomized, double-blind, placebo-controlled 26-week trial to evaluate the efficacy and safety of dimebon in patients with moderate-to-severe Alzheimer's disease. ClinicalTrialsgov [<http://clinicaltrialsgov];> 2009.

171. Anon. [Public title] Safety and efficacy study evaluating dimebon in patients with mild to moderate Alzheimer's disease on donepezil CONCERT; [Scientific title] CONCERT: A phase 3 multicenter, randomized, placebo-controlled, double-bind twelve-month safety and efficacy study evaluating dimebon in patients with mild-to-moderate Alzheimer's disease on donepezil. ClinicalTrialsgov [<http://clinicaltrialsgov];> 2009.

172. Anon. [Public title] A safety and efficacy study evaluating dimebon (Latrepirdine) in patients with moderate to severe Alzheimer's disease (CONTACT); [Scientific title] CONTACT: a phase 3 multicenter, randomized, double-blind, placebo-controlled, six-month safety and efficacy study of dimebon in patients with moderate-to-severe Alzheimer's disease. ClinicalTrialsgov [<http://clinicaltrialsgov];> 2009.

173. Anon. [Public title] A phase 3 study to evaluate the safety and tolerability of dimebon patients with mild to Moderate Alzheimer's Disease; [Scientific title] A Phase 3, Multi-Center, Randomized, Double-Blind Placebo-Controlled Study To Evaluate The Safety And Tolerability Of Dimebon (PF-01913539) For Up To 26-Weeks In Patients With Mild To Moderate Alzheimer's Disease. ClinicalTrialsgov [<http://clinicaltrialsgov];> 2009.

174. Medivation I. A Safety and Efficacy Study of Oral Dimebon in Patients With Mild-To-Moderate Alzheimer's Disease (CONNECTION). https://clinicaltrials.gov/ct2/show/NCT00675623?term=NCT0067562. ClinicalTrialsgov [<http://clinicaltrialsgov];> 2008.

175. Pfizer. A Study Of Oral PF-01913539 In Patients With Mild To Moderate Alzheimer's Disease. https://clinicaltrials.gov/ct2/show/NCT01066481?term=NCT0106648. ClinicalTrialsgov [<http://clinicaltrialsgov];> 2010.

176. Pfizer. Prevention of Cognitive Decline in Alzheimer's Disease by Ingested Interferon Alpha. https://clinicaltrials.gov/ct2/show/NCT00031018. 2002.

177. Grimaldi LM, Zappala G, Iemolo F, et al. A pilot study on the use of interferon beta-1a in early Alzheimer's disease subjects. *J Neuroinflammation*. 2014;**11**:30.

178. Adair JC, Knoefel JE, Morgan N. Controlled trial of N-acetylcysteine for patients with probable Alzheimer's disease. *Neurology*. 2001;**57**:1515-7.

179. Hoffmann-La Roche. MAyflOwer RoAD Study: A Study of RO4602522 in Patients With Moderate Severity Alzheimer Disease on Background Alzheimer Disease Therapy. https://clinicaltrials.gov/ct2/show/NCT01677754?term=NCT0167775. ClinicalTrialsgov [<http://clinicaltrialsgov];> 2012.

180. Lowe D. A Multicenter, Randomized, Double-Blind, Placebo-Controlled Study to Evaluate Safety and Efficacy of MEM 1003 in Patients With Mild to Moderate Alzheimer’s Disease. ClinicalTrialsgov [<http://clinicaltrialsgov];> 2005.

181. Anon. [Public title] Preliminary efficacy and safety study of ST101 in Alzheimer's disease; [Scientific title] A double-blind placebo-controlled preliminary study of the efficacy, safety and tolerability of ST101 tablets in the treatment of Alzheimer's disease. ClinicalTrialsgov [<http://clinicaltrialsgov];> 2009.

182. Anon. Preliminary efficacy and safety study of ST101 plus aricept in Alzheimer's disease. ClinicalTrialsgov [<http://clinicaltrialsgov];> 2009.

183. Molloy DW, Standish TI, Zhou Q, Guyatt G. A multicenter, blinded, randomized, factorial controlled trial of doxycycline and rifampin for treatment of Alzheimer's disease: the DARAD trial. *Int J Geriatr Psychiatry*. 2013;**28**:463-70.

184. Loeb MB, Molloy DW, Smieja M, et al. A randomized, controlled trial of doxycycline and rifampin for patients with Alzheimer's disease. *J Am Geriatr Soc*. 2004;**52**:381-7.

185. Anon. [Public title] Doxycycline and rifampin for Alzheimer's disease; [Scientific title] Multi-centre, blinded, randomised, controlled trial comparing different regimens of the antibiotics doxycycline and rifampin for treatment of Alzheimer's disease. ISRCTN Register [<http://wwwcontrolled-trialscom];> 2006.

186. Alvarez XA, Cacabelos R, Sampedro C, et al. Combination treatment in Alzheimer's disease: results of a randomized, controlled trial with cerebrolysin and donepezil. *Curr Alzheimer Res*. 2011;**8**:583-91.

187. Alvarez XA, Cacabelos R, Laredo M, et al. A 24-week, double-blind, placebo-controlled study of three dosages of Cerebrolysin in patients with mild to moderate Alzheimer's disease. *Eur J Neurol*. 2006;**13**:43-54.

188. Panisset M, Gauthier S, Moessler H, Windisch M. Cerebrolysin in Alzheimer's disease: a randomized, double-blind, placebo-controlled trial with a neurotrophic agent. *J Neural Transm*. 2002;**109**:1089-104.

189. Alvarez A, Laredo M, Sampedro C, et al. Results Of A Randomized, Double-blind, Placebo-controlled Trial With The Neurotrophic Compound Cerebrolysin In Alzheimer's Disease Patients. Neurobiology of Aging; 2004. p. 209.

190. Ever Neuro Pharma GmbH. Cerebrolysin Compared to Donepezil in Patients With Mild to Moderate Dementia of Alzheimer's Type (DAT). https://clinicaltrials.gov/ct2/show/NCT01822951?term=dat+cerebrolysin&rank=1. ClinicalTrialsgov [<http://clinicaltrialsgov];> 2013.

191. Ruether E, Husmann R, Kinzler E, et al. A 28-week, double-blind, placebo-controlled study with Cerebrolysin in patients with mild to moderate Alzheimer’s disease. *International Clinical Psychopharmacology* 2001;**16**:253-63.

192. Anon. A randomized, double-blind, placebo-controlled, parallel group, phase 2 study to evaluate the safety and efficacy of FK962 in subjects with mild to moderate Alzheimer's disease. ClinicalTrialsgov [<http://clinicaltrialsgov];> 2004.

193. VIVUS I. VI-1121 for the Treatment Alzheimer's Disease (AD-201). https://clinicaltrials.gov/ct2/show/NCT01428362?term=NCT0142836. ClinicalTrialsgov [<http://clinicaltrialsgov];> 2011.

194. Thal LJ, Grundman M, Berg J, et al. Idebenone treatment fails to slow cognitive decline in Alzheimer's disease. *Neurology*. 2003;**61**:1498-502.

195. Gutzmann H, Kuhl KP, Hadler D, Rapp MA. Safety and efficacy of idebenone versus tacrine in patients with Alzheimer's disease: results of a randomized, double-blind, parallel-group multicenter study. *Pharmacopsychiatry*. 2002;**35**:12-8.

196. Toyama Chemical Co. L. Efficacy and Safety of T-817MA in Patients With Mild to Moderate Alzheimer's Disease. https://clinicaltrials.gov/ct2/show/NCT00663936?term=NCT0066393. ClinicalTrialsgov [<http://clinicaltrialsgov];> 2008.
