# Supplementary material for: Older patients are still under-represented in clinical trials of Alzheimer’s disease
Source: Alzheimers Res Ther. 2016 Aug 12;8:32. doi: 10.1186/s13195-016-0201-2 (PMC4982205; doi:10.1186/s13195-016-0201-2)
Supplement: Additional file 4: — Main characteristics of trials included in the age analyses (79). (DOCX 30 kb) [file 13195_2016_201_MOESM4_ESM.docx]

**Additional file 4 – Main characteristics of trials included in the age analyses (79)**

| **First author/year/**  **trial name** | **Phase** | **Investigational drug** | **Size of the study^*^** | **Target population** | **Countries** | **Age at entry** | **Mean age** | **Range and/or SD** |
| --- | --- | --- | --- | --- | --- | --- | --- | --- |
| Saumier 2009 and Aisen 2011 (ALPHASE) | III | Tramiprosate | 1052 | Probable, mild to moderate | USA, Canada | 50+ | 73.9 | 48-94 |
| Gilman 2005 | II | AN1792 | 372 | Probable, mild to moderate | USA, Europe | 50-85 | 72.1 | 7.8 |
| Sunovion 2006 | II | Lornoxicam | 219 | Mild to moderate | USA | 55+ | 75.6 |  |
| Mohs 2009 | II/III | Atomoxetine | 92 | Probable, mild to moderate | USA | 55+ | 77.4 | 7.4 |
| Van Gool 2001 | III | Hydroxychloroquine | 168 | Probable, mild | The Netherlands | NR | 70.6 | 8.4 |
| Thal 2003 | NR | Idebenone | 536 | Probable, mild to moderate | USA | 50+ | 75.4 | 8.2 |
| Gutzmann 2002 | III | Idebenone | 203 | Probable, mild to moderate | Germany | 40-90 | 71.3 | 44-90 (9.9) |
| Doody 2013 | III | Semagacestat | 1537 | Probable, mild to moderate | North and South America, Australia, Europe, Asia, South Africa | 55+ | 73.2 | 8.2 |
| Anon 2008  (IDENTITY II) | III | Semagacestat | 1108 | Mild to moderate | North and South America, Australia, Europe, Asia | 55+ | 73.2 | 8.0 |
| Fleisher 2008 | II | Semagacestat | 51 | Probable, mild to moderate | USA | 50+ | 69.4 | 9.1 |
| Siemers 2006 | II | Semagacestat | 70 | Probable, mild to moderate | USA | 50+ | 70.3 | 7.9 |
| Doody 2014 (EXPEDITION 1) | III | Solanezumab | 1012 | Probable, mild to moderate | North and South America, Japan | 55+ | 74.7 | 8.0 |
| Doody 2014 (EXPEDITION 2) | III | Solanezumab | 1040 | Probable, mild to moderate | Europe, USA, Australia, Asia | 55+ | 72.5 | 7.9 |
| Salloway 2009 | II | Bapineuzumab | 234 | Probable, mild to moderate | USA | 50-85 | 69.1 | 9.0 |
| Salloway 2014  (APO E4 carriers) | III | Bapineuzumab | 1090 | Probable, mild to moderate | USA | 55-88 | 72.1 | 8.2 |
| Salloway 2014  (APO E4 noncarriers) | III | Bapineuzumab | 1114 | Probable, mild to moderate | USA, Canada, Europe | 55-88 | 72.7 | 9.6 |
| Anon 2008 | III | IGIV 10% | 383 | Probable, mild to moderate | USA, Canada | 50-89 | 70.3 | 9.3 |
| Craft 2012  (SNIF 120) | II | Intranasal insulin | 111 | Probable, mild to moderate and mild cognitive impairment | USA | 55+ | 72.3 | 8.9 |
| Claxton 2014  (SNIF LONG) | II | Intranasal insulin | 60 | Probable, mild to moderate and mild cognitive impairment | USA | 50-89 | 72.0 | 8.4 |
| Rosenbloom 2014 | II | Insulin glulisine | 12 | Probable, mild to moderate | USA | 65-85 | 72.0 | 8.2 |
| Sato 2011 | II | Pioglitazone | 42 | Probable, mild | Japan | NR | 77.5 | 6.3 |
| Hanyu 2008 | II | Pioglitazone | 32 | Probable, mild to moderate | Japan | NR | 77.2 | 6.0 |
| Gold 2010  (REFLECT-1) | III | Rosiglitazone | 581 | Probable, mild to moderate | Europe, Asia, North and South America, New Zealand | 50-90 | 72.3 | 8.3 |
| Harrington 2006 (REFLECT-2) | III | Rosiglitazone | 1496 | Probable, mild to moderate | Europe, Asia, North and South America | 50-90 | 74.1 | 50-91 |
| Harrington 2006 (REFLECT-3) | III | Rosiglitazone | 1485 | Probable, mild to moderate | Europe, Asia, Africa, North America | 50-90 | 73.2 | 50-90 |
| AD 2000 | III | Aspirin | 310 | Probable, mild to moderate | UK | NR | 74.7 | 7.1 |
| Pasqualetti 2009 | NR | Ibuprofen | 132 | Probable, mild to moderate | Italy | 65+ | 73.9 | 7.5 |
| De Jong 2008 | III | Indomethacin | 51 | Probable, mild to moderate | The Netherlands | NR | 72.5 | 7.9 |
| Aisen 2002 | II | Nimesulide | 40 | Probable, mild to moderate | USA | NR | 73.5 | 9.1 |
| Soininen 2007 | III | celecoxib | 425 | Probable, mild to moderate | USA, Australia, Europe, | 50+ | 73.6 | 8.1 |
| Aisen 2003  (NSAID study) | II/III | rofecoxib | 351 | Probable, mild to moderate | USA | 50+ | 73.9 | 7.6 |
| Reines 2004 | NR | rofecoxib | 692 | Possible or probable, mild to moderate | USA | 50+ | 75.5 | 8.5 |
| Aisen 2002 (Alzheimer’s Disease Cooperative Study) | III | prednisone | 138 | Probable, mild to moderate | USA | 50+ | 72.9 | 7.6 |
| Wolkowitz 2003 | NR | dehydroepiandrosterone | 58 | Probable, mild to moderate | USA | 55+ | 76.4 | 7.4 |
| Lu 2006 | NR | testosterone | 18 | Probable, mild to moderate | USA | 50+ | 69.8 | 8.5 |
| Pomara 2002 | II | mifepristone | 9 | Probable, mild to moderate | USA | NR | 72.0 | 10.8 |
| Yoon 2003 | NR | Hormon-replacement therapy | 55 | Probable, mild to moderate | Korea | NR | 70.1 | 53-85 (6.8) |
| Wang 2002 | NR | Hormon-replacement therapy | 50 | Probable, mild to moderate | Taiwan | 60+ | 71.8 | 9.0 |
| Mulnard 2000 (Alzheimer’s Disease Cooperative Study) | NR | Hormon-replacement therapy | 120 | Probable, mild to moderate | USA | 60+ | 75.1 | 56-91 (11.1) |
| Henderson 2000 | NR | Hormon-replacement therapy | 42 | Probable, mild to moderate | USA | NR | 77.5 | 5.5 |
| Wharton 2011 | NR | Hormon-replacement therapy | 43 | Mild to moderate | USA | 55-85 | 76.8 | 7.6 |
| Stanford University 2006 | II | Raloxifene | 42 | Probable, mild to moderate | USA | 60+ | 76.0 | 4.8 |
| Bowen and Powers 2014  (ALADDIN) | II | Leuprolide acetate | 109 | Probable, mild to moderate | USA | 65+ | 77.0 | 65-93 (6.0) |
| Marek 2014 | II | ABT-384 | 267 | Probable, mild to moderate | Europe, Russia, South Africa | 55-90 | 72.0 | 8.5 |
| Schwam 2014 | II | PF-04447943 | 191 | Probable, mild to moderate | North and South America, Czech  Republic | 55-85 | 73.5 | 54-85 (7.8) |
| Thal 2000 | NR | Lu25-109 | 496 | Probable, mild to moderate | USA | 45+ | 75.5 | 47-95 |
| Grimaldi 2014  (REAL) | II | Interferon beta 1a | 42 | Mild | Italy | 50-75 | 63.7 | 7.9 |
| Burstein 2014 | II | TTP488 | 399 | Probable, mild to moderate | USA | 50+ | 72.9 | 9.1 |
| Doody 2008 | II | Dimebon | 183 | Probable, mild to moderate | Russia | 50+ | 68.3 | 9.0 |
| Loeb 2004 | II | Doxycycline and rifampin | 101 | Probable, mild to moderate | Canada | NR | 75.5 | 65-99 (8.0) |
| Molloy 2013  (DARAD) | III | Doxycycline and rifampin | 406 | Probable, mild to moderate | Canada | 50-99 | 78.5 | 7.1 |
| Grove 2014 and Anon 2009 | II | GSK239512 | 196 | Probable, mild to moderate | Europe, Korea, Russia | 50+ | 71.8 | 50-97 (8.7) |
| Egan 2012 | II | MK0249 | 144 | Probable, mild to moderate | USA | 55+ | 74.1 | 7.9 |
| Haig 2014 | II | ABT 288 | 242 | Probable, mild to moderate | Russia, Ukraine | 55-90 | 70.2 | 8.3 |
| Carlson 2002 | NR | Nizatidine | 51 | Possible or probable (severity not reported) | USA | 65+ | 80.7 | 67-96 |
| Lenz 2015 | II | ABT 089 | 337 | Probable, mild to moderate | USA | 55-90 | 75.6 | 8.0 |
| Kim 2009 | II | Varenicline | 66 | Probable, mild to moderate | Korea | 55-85 | 72.7 | 55-85 (7.4) |
| Ritchie 2003 | II | Clioquinol | 36 | Probable, mild to moderate | Australia, Europe | NR | 72.5 | 8.4 |
| Piette 2011 | II | Masitinib | 34 | Probable, mild to moderate | France | 50+ | 75.1 | 11.8 |
| Wilcock 2008 | II | Tarenflurbil | 210 | Probable, mild to moderate | Canada, UK | 55+ | 74.6 | 7.8 |
| Green 2009 | III | Tarenflurbil | 1684 | Probable, mild | USA | 55+ | 76.4 | 53-100 (8.4) |
| Sano 2011  (CLAPS) | III | Simvastatine | 406 | Probable, mild to moderate | USA | 50+ | 74.6 | 9.3 |
| Feldman 2010 and Jones 2008 (LEADe) | III | Atorvastatine | 641 | Probable, mild to moderate | USA | 50-90 | 73.6 | 8.4 |
| Sparks 2006  (ADCTL) | II | Atorvastatine | 67 | Probable and possible, mild to moderate | USA | 51+ | 78.5 | 7.0 |
| Wilkinson 2014 (LADDER) | II | Lu AE58054 | 278 | Probable, mild | Australia, Canada, Europe | 50+ | 74.5 | 7.4 |
| Maher-Edwards 2010 | II | SB 742457 | 198 | Probable, mild to moderate | Europe, Russia, Chile | 50-85 | 71.2 | 7.8 |
| Maher-Edwards 2010 | II | SB 742457 | 371 | Probable, mild to moderate | Europe, South America, Asia, South Africa, New Zealand, Russia | 50-85 | 69.8 | 8.6 |
| Alvarez 2004 and 2006 | II | Cerebrolysin | 279 | Probable, mild to moderate | Spain | 50+ | 73.6 | 8.9 |
| Alvarez 2011  (COMBI) | II | Cerebrolysin | 217 | Probable, mild to moderate | Spain | 51+ | 75.2 | 8.3 |
| Panisset 2002 | II | Cerebrolysin | 192 | Probable, mild to moderate | Canada | 60+ | 74.2 | 59-91 (6.2) |
| Ruether 2001 | II | Cerebrolysin | 149 | Probable, mild to moderate | Germany and Austria | 50-85 | 73.0 | 7.8 |
| Sevigny 2008 | II | MK 677 | 563 | Probable, mild to moderate | USA | 50+ | 76.0 | 8.6 |
| Wischik 2015 | II | Trx0014 | 321 | Probable, mild to moderate | UK, Singapore | NR | 73.8 | 9.0 |
| JSW Lifescience 2010 | II | Lornoxicam | 219 | Probable, mild to moderate | Europe | 50-85 | 74.1 | 7.5 |
| Orion 2011 | II | ORM 12741 | 100 | Probable, mild to moderate | Europe | 55-90 | 72.0 | 8.2 |
| Shanghai Greenvalley 2011 | II | Sodium oligo-mannurate | 255 | Probable, mild to moderate | China | 50-85 | 70.3 | 8.2 |
| Lovestone 2015 (ARGO) | II | Tideglusib | 306 | Probable, mild to moderate | Europe | 50-85 | 71.5 | 8.3 |
| Sanofi 2011 | II | SAR110894D | 291 | Probable, mild to moderate | North America, Europe, Australia | 55+ | 72.6 | 8.2 |
| Frolich 2011 (SIROCCO) | II | AZD 3480 | 567 | Probable, mild to moderate | Europe, Canada | 60-85 | 73.6 | 6.4 |

SD standard deviation.

^*^  The number of participants randomized in each trial. Overall, 27,290 participants were randomized in the 79 trials. Ages were stated for 26,845 of these.
